# Ambient Synthesis of Cyclohexanone Oxime via In Situ Produced Hydrogen Peroxide over Cobalt‐Based Electrocatalyst

**DOI:** 10.1002/advs.202413475

**Published:** 2024-12-17

**Authors:** Hui Xu, Meng Jin, Shengbo Zhang, Xinyuan Zhang, Min Xu, Yunxia Zhang, Guozhong Wang, Haimin Zhang

**Affiliations:** ^1^ Key Laboratory of Materials Physics Centre for Environmental and Energy Nanomaterials Anhui Key Laboratory of Nanomaterials and Nanotechnology Institute of Solid State Physics HFIPS Chinese Academy of Sciences Hefei 230031 China; ^2^ University of Science and Technology of China Hefei 230026 China

**Keywords:** ammoximation, Co nanoparticles, Co single atoms, H_2_O_2_ electrosynthesis, TS‐1 heterogeneous catalyst

## Abstract

Cyclohexanone oxime, a critical precursor for nylon‐6 production, is traditionally synthesized via the hydroxylamine method under industrial harsh conditions. Here is present a one‐step electrochemical integrated approach for the efficient production of cyclohexanone oxime under ambient conditions. This approach employed the coupling of in situ electro‐synthesized H_2_O_2_ over a cobalt (Co)‐based electrocatalyst with the titanium silicate‐1 (TS‐1) heterogeneous catalyst to achieve the cyclohexanone ammoximation process. The cathode electrocatalyst is consisted of atomically dispersed Co sites and small Co nanoparticles co‐anchored on carboxylic multi‐walled carbon nanotubes (CoSAs/SNPs‐OCNTs), which delivered superior electrocatalytic activity toward the two‐electron oxygen reduction reaction (2e^−^ ORR) with high‐efficient H_2_O_2_ production in 0.1 m sodium phosphate (NaPi). Theoretical calculations revealed that the introduction of Co nanoparticles effectively optimized the binding strength of ^*^OOH species on Co atomic sites, thus facilitating the 2e^−^ ORR. The subsequent tandem catalytic system achieved a high cyclohexanone conversion of 71.7% ± 1.1% with a cyclohexanone oxime selectivity of 70.3% ± 0.6%. In this system, the TS‐1 catalyst effectively captured the ^*^OOH intermediate and activated the in situ generated H_2_O_2_ to form Ti‐OOH species, which promoted the formation of hydroxylamine and thereby enhanced the oxime production performance.

## Graphical abstract

A high‐efficiency 2e^−^ ORR electrocatalyst composed of atomically dispersed Co sites and ultrafine Co nanoparticles is fabricated and exhibited exceptional electrocatalytic performance toward H_2_O_2_ synthesis, further coupling with TS‐1 catalyst in an integrated electrochemical system to achieve sustainable synthesis of cyclohexanone oxime under ambient conditions.

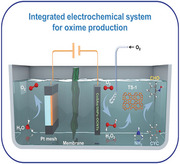

## 1 Introduction

Cyclohexanone oxime (CHO) is a critical precursor for caprolactam production via Beckmann rearrangement, which is an upstream industry for the synthesis of nylon‐6.^[^ ^1^ ^]^ As a significant feedstock of fiber and textile manufacturing, global production of nylon‐6 is predicted to reach ≈9 million tons per year by 2026, thus the demand for CHO will concurrently rise.^[^ ^2^ ^]^ Currently, the majority of CHO is manufactured by the hydroxylamine (NH_2_OH) method, in which the nitrogen oxides (NO_x_) are first reduced to NH_2_OH by reductants (H_2_ or SO_2_) and then the formed NH_2_OH reacts non‐catalytically with cyclohexanone (CYC) to generate CHO (Figure S1a, Supporting Information).^[^ ^3^ ^]^ This process is operated under strongly acidic reaction conditions with noble metal palladium (Pd) catalysts and produces ammonium sulfate as the by‐product, thereby raising great concerns about safety, cost, and sustainability.^[^ ^3^, ^4^ ^]^ A one‐step strategy was developed to address the issues mentioned above, namely the cyclohexanone ammoximation process, which used titanium silicate‐1 (TS‐1) as the catalyst, with CYC, ammonia (NH_3_), and hydrogen peroxide (H_2_O_2_) as reactants (Figure S1b, Supporting Information).^[^ ^5^ ^]^ In this route, NH_2_OH is synthesized via the oxidation of NH_3_ by H_2_O_2_ on Ti^IV^ sites in the titanosilicate framework and subsequently attacks CYC to generate CHO.^[^ ^6^ ^]^ In spite of the ammoximation process delivers brilliant catalytic selectivity and efficiency toward oxime production, the excess addition of H_2_O_2_ is indispensable owing to its low stability under harsh ammoximation reaction conditions including high temperature and pH, thereby causing extra costs. Also, such commercial H_2_O_2_ is usually manufactured by the energy‐intensive anthraquinone process with substantial costs in tranportation, handling, and storage stage.^[^ ^7^ ^]^ To solve the above issues, recently, Hutchings's group reported that the combination of in situ generated H_2_O_2_ on gold‐palladium (AuPd) alloyed nanoparticles and TS‐1 catalyst achieved comparable CHO yield to the current industrial process (Figure S1c, Supporting Information).^[^ ^8^ ^]^ However, this strategy still required precious metal catalysts to catalyze the direct synthesis of H_2_O_2_ by the mixture of H_2_ and O_2_ under high temperature and pressure. Therefore, developing alternative approaches for in situ high‐efficiency synthesis of H_2_O_2_ under ambient conditions, combined with TS‐1 catalyst, is highly needed for green and efficient synthesis of CHO.

Recently, the electrochemical synthesis strategy via the two‐electron oxygen reduction reaction (2e^−^ ORR) has been developed for the decentralized and on‐site production of H_2_O_2_. Such in situ electro‐synthesized H_2_O_2_ can be easily coupled with TS‐1 catalyst to form Ti‐OOH active sites, which can further upgrade organics to value‐added counterparts.^[^ ^9^ ^]^ For example, Kwak's group established an integrated photo‐electro‐heterogeneous catalytic system, achieving the direct propylene epoxidation to propylene oxide by the in situ generated H_2_O_2_ and TS‐1 heterogeneous catalyst.^[^ ^9a^ ^]^ Lu et al. reported the tandem route with mesoporous carbon materials as the 2e^−^ ORR electrocatalyst to effectively produce H_2_O_2_, which was subsequently coupled with TS‐1 catalyst to achieve selective oxidation of ethylene to ethylene glycol with a remarkable selectivity of ≈99.99%.^[^ ^9b^ ^]^ Wang's group developed an interfacial electrochemical‐chemical reaction system utilizing high concentration of H_2_O_2_ formed at the interface and TS‐1 catalyst in the solid electrolyte to deliver 583 µmol h^−1^ production rate of ethylene glycol.^[^ ^9c^ ^]^ Based on the above discussions, it is highly feasible and desirable to couple in situ electrogenerated H_2_O_2_ with TS‐1 catalyst to efficiently synthesize the oxime under ambient conditions (Figure S1d, Supporting Information).

Herein, we report an integrated electrochemical route to highly efficiency synthesize CHO in a green and cost‐effective manner. The cathode electrocatalyst, composed of atomically dispersed Co sites and small Co nanoparticles (NPs) co‐anchored on carboxyl‐functionalized multi‐walled carbon nanotubes (denoted as CoSAs/SNPs‐OCNTs), demonstrated exceptional electrocatalytic performance toward 2e^−^ ORR. Specifically, this catalyst afforded a positive onset potential of 0.73 V (vs RHE) and a maximum H_2_O_2_ selectivity of 95.9% in 0.1 m sodium phosphate (NaPi). The CoSAs/SNPs‐OCNTs also exhibited high electrocatalytic activity for H_2_O_2_ production, delivering a high yield rate of 567 ± 25 mmol g_cat_^−1^ h^−1^ with a faradaic efficiency (FE) of 88.9% ± 6.3% at 0.2 V (vs RHE), providing the necessary premise for the subsequent ammoximation process. Theoretical calculations disclosed that the interaction between Co‐N_4_ atomic sites and Co NPs can efficiently tailor the electronic structure of Co‐N_4_ sites and moderate the binding energy of the ^*^OOH intermediate, thereby boosting the electrocatalytic performance toward 2e^−^ ORR. The in situ electrogenerated H_2_O_2_ was then coupled with the TS‐1 catalyst, resulting in a high CYC conversion of 71.7% ± 1.1% with a CHO selectivity of 70.3% ± 0.6%. Control experiments confirmed that NH_2_OH was the key N‐containing species generated during the reaction, which rapidly reacted with CYC to form CHO through a nucleophilic addition‐elimination process. The strategy developed in this work can also be extended to other ketones and aldehydes, demonstrating its broad applicability.

## 2 Results and Discussion

### 2.1 Synthesis and Structural Characterization of CoSAs/SNPs‐OCNTs

**Figure 1:**
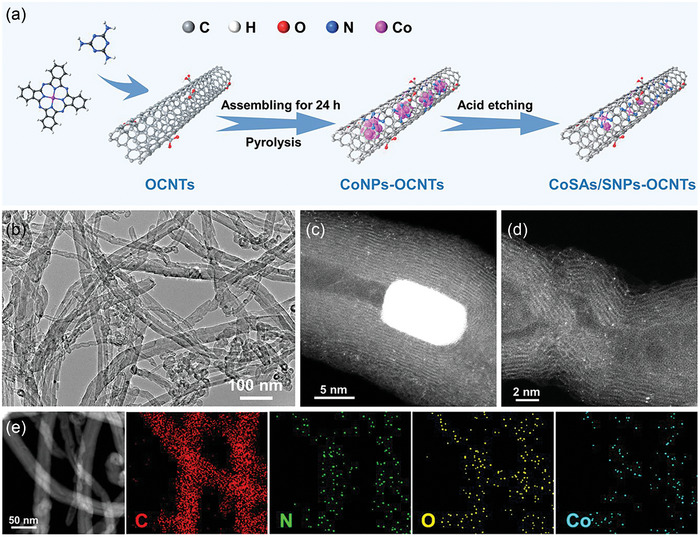
a) Schematic illustration of the fabrication process of CoSAs/SNPs‐OCNTs. b) TEM and c,d) aberration‐corrected HAADF‐STEM images of CoSAs/SNPs‐OCNTs. e) HAADF‐STEM image and corresponding elemental mapping images of CoSAs/SNPs‐OCNTs.

The synthetic procedure of CoSAs/SNPs‐OCNTs is schematically depicted in **Figure** **1**a. Specifically, cobalt phthalocyanine (CoPc) was first assembled on the surface of OCNTs by the π‐π interaction^[^ ^10^ ^]^ and melamine was introduced as the nitrogen source. Subsequently, high‐temperature pyrolysis was conducted under Ar atmosphere followed by acid‐etching treatment to acquire the CoSAs/SNPs‐OCNTs. For comparison, the large‐sized Co nanoparticles sample (CoNPs‐OCNTs) was also synthesized without the acid‐etching treatment. The scanning electron microscopy (SEM) images of CoSAs/SNPs‐OCNTs and CoNPs‐OCNTs display well‐maintained nanotube morphology (Figure S2, Supporting Information). Further transmission electron microscopy (TEM) image of CoNPs‐OCNTs confirms the formation of large‐sized NPs with particle sizes of 30–100 nm embedded on OCNTs (Figure S3, Supporting Information). The high‐resolution TEM (HR‐TEM) image displays the lattice spacing of 0.21 and 0.34 nm corresponding to the (111) plane of metallic Co and the (002) plane of graphitic carbon, respectively. While, as for the CoSAs/SNPs‐OCNTs sample, the TEM image manifests a few small‐sized NPs embedded on OCNTs (Figure 1b) and the Co content is drastically decreased from 5.11 wt.% in CoNPs‐OCNTs to 0.19 wt.%, demonstrating the removal of large‐sized Co NPs after acid‐etching process. In order to precisely characterize the existence form of Co species in CoSAs/SNPs‐OCNTs, the aberration‐corrected high‐angle annular dark‐field scanning transmission electron microscopy (HAADF‐STEM) was further employed, which explicitly revealed the coexistence of ultrafine Co NPs and atomically dispersed Co sites (Figure 1c,d). The small‐sized Co NPs is ≈10 nm with enriched atomic Co sites surrounded (Figure S4, Supporting Information). The HADDF‐STEM image and corresponding energy‐dispersive X‐ray spectroscopy (EDS) elemental mapping images are displayed in Figure 1e, which exhibit the homogeneous distributions of C, N, O, and Co elements throughout CoSAs/SNPs‐OCNTs.

**Figure 2:**
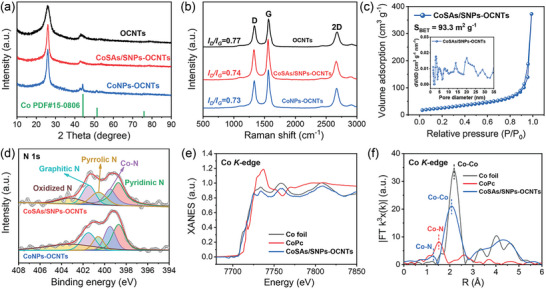
a) XRD patterns and b) Raman spectra of OCNTs, CoNPs‐OCNTs and CoSAs/SNPs‐OCNTs. c) Nitrogen adsorption–desorption isotherms of CoSAs/SNPs‐OCNTs. Inset: corresponding pore size distribution curve. d) High‐resolution N 1s XPS spectrum of CoNPs‐OCNTs and CoSAs/SNPs‐OCNTs. Co K‐edge e) XANES spectra and (f) k^3^‐weighted FT‐EXAFS spectra of CoSAs/SNPs‐OCNTs and references.

The crystalline structures of the obtained samples were further studied by the X‐ray diffraction (XRD) analysis. The XRD patterns of CoNPs‐OCNTs displays the peaks at 44.2, 51.6 and 75.9° corresponding to the (111), (200), and (220) lattice planes of cubic Co phase (**Figure** **2**a), respectively, indicating the formation of metallic Co NPs. By contrast, these peaks are absent in the CoSAs/SNPs‐OCNTs, with only the peaks related to graphitic carbon in OCNTs being detected, further indicative of the removal of large‐sized Co NPs. The Raman spectroscopy was further employed to investigate the carbon structure of this set of samples. As exhibited in Figure 2b, two bands located at ≈1330 and 1560 cm^−1^ were detected, attributing to the disordered carbon (D band) and the graphitic carbon (G band), respectively.^[^ ^11^ ^]^ These samples displayed similar relative intensity ratios of the D to G bands (*I_D_/I_G_*), indicating their prominent graphitic structures. Additionally, a well‐defined 2D band was also observed at ≈2670 cm^−1^, further suggesting the high degree of graphitization in these samples. The specific surface area and porous structure of CoSAs/SNPs‐OCNTs were investigated by the N_2_ adsorption–desorption isotherms. As exhibited in Figure 2c, the N_2_ adsorption–desorption isotherms of CoSAs/SNPs‐OCNTs demonstrate typical type IV characteristics with an H_3_‐typed hysteresis loop, indicative of mesoporosity.^[^ ^12^ ^]^ The pore size distribution for CoSAs/SNPs‐OCNTs was further analyzed using the density functional theory (DFT) method, revealing a predominance of mesopores ranging from 2–35 nm in diameter with a large pore volume of 0.32 cm^3^ g^−1^ (inset of Figure 2c). The formation of this mesoporous structure is likely due to the acid‐etching treatment, which can remove Co‐related large NPs, thereby creating mesopores.^[^ ^13^ ^]^ The Brunauer–Emmett–Teller (BET) specific surface area of CoSAs/SNPs‐OCNTs obtained from N_2_ adsorption–desorption isotherms was measured to be 93.3 m^2^ g^−1^, favoring the exposure of active sites and facilitating the mass transport during electrocatalysis.

The X‐ray photoelectron spectroscopy (XPS) was performed to examine the elemental composition and chemical state of as‐prepared catalysts. The XPS survey spectrum of CoSAs/SNPs‐OCNTs reveals the presence of C, N, and O elements (Figure S5a, Supporting Information). The absence of Co signal in the spectrum is attributed to its low content in the sample. The high‐resolution C 1s spectra (Figure S5b, Supporting Information) can be divided into three carbon species, including C─C/C═C (284.8 eV), C─N/C─O (285.7 eV) and O─C═O (288.8 eV).^[^ ^14^ ^]^ And the high‐resolution N 1s spectra can be deconvoluted into five peaks, namely pyridinic N (398.7 eV), Co─N (399.5 eV), pyrrolic N (400.6 eV), graphitic N (401.5 eV) and oxidized N (403.2 eV),^[^ ^15^ ^]^ as depicted in Figure 2d. Additionally, the deconvolution of the O 1s spectra (Figure S5c, Supporting Information) reveal the presence of two distinct peaks at 531.6 and 533.2 eV, attributing to oxygen doubly bound to carbon (C═O) and oxygen singly bound to carbon (C─O),^[^ ^16^ ^]^ respectively, confirming the existence of abundant oxygen functional groups on the surface of CoSAs/SNPs‐OCNTs. The high‐resolution Co 2p spectrum of CoNPs‐OCNTs can be deconvoluted to Co^0^ (778.5 and 793.8 eV), Co 2p_3/2_ (781.0 eV), Co 2p_1/2_ (797.1 eV) and satellite peaks (786.5 and 803.7 eV) (Figure S5d, Supporting Information), manifesting the coexistence of metallic Co and oxidized Co.^[^ ^17^ ^]^ The detection of oxidized Co in the Co 2p XPS spectrum is attributed to the oxidation of surface metallic Co upon exposure to the ambient air. Since the low content of Co element is undetectable in the XPS characterization (Figure S5d, Supporting Information), the X‐ray absorption spectroscopy (XAS) was further employed to analyze the structure details of Co species in CoSAs/SNPs‐OCNTs. From Co K‐edge X‐ray absorption near‐edge structure (XANES) spectra, the absorption edge of CoSAs/SNPs‐OCNTs was found to be similar to that of Co foil (Figure 2e), implying the predominance of metallic Co in CoSAs/SNPs‐OCNTs. The Fourier transformation extended X‐ray absorption fine structure (FT‐EXAFS) spectrum of CoSAs/SNPs‐OCNTs manifests two peaks at 1.3 and 2.1 Å in R space (Figure 2f), which are close to Co─N bond and Co─Co bond, respectively.^[^ ^17^, ^18^ ^]^ Therefore, the above characterizations verify the presence of both Co single atoms (SAs) and Co NPs in the CoSAs/SNPs‐OCNTs. These findings are consistent with the observations made using the aberration‐corrected HAADF‐STEM and are analogous to previous reports describing simultaneous observation of SAs and NPs in similar contexts in the literatures.^[^ ^17^, ^18^, ^19^ ^]^

### 2.2 Electrocatalytic Performance toward 2e^−^ ORR

**Figure 3:**
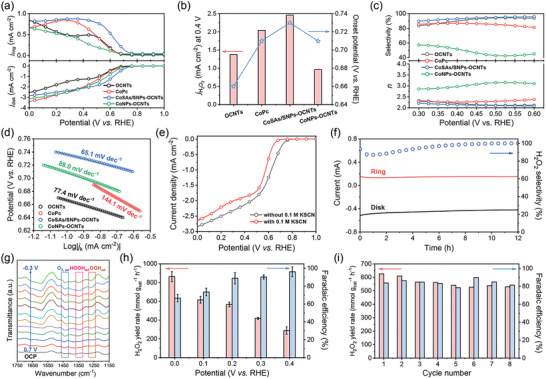
a) LSV curves of OCNTs, CoPc, CoSAs/SNPs‐OCNTs and CoNPs‐OCNTs obtained at 1600 rpm under 10 mV s^−1^ in O_2_‐saturated 0.1 m NaPi. b) Comparison of H_2_O_2_ current density (*j_H2O2_*) at 0.4 V (vs RHE) and onset potential for OCNTs, CoPc, CoSAs/SNPs‐OCNTs, and CoNPs‐OCNTs. c) Calculated H_2_O_2_ selectivity and electron transfer number (*n*) based on LSV data. d) Tafel slopes of various catalysts. e) LSV curves of CoSAs/SNPs‐OCNTs in O_2_‐saturated 0.1 m NaPi with and without 0.1 m KSCN. f) Stability test of CoSAs/SNPs‐OCNTs at 0.4 V (vs RHE) in 0.1 m NaPi for 12 h. g) In situ ATR‐SEIRAS spectra collected on CoSAs/SNPs‐OCNTs catalyst in O_2_‐saturated 0.1 m NaPi at different applied potentials. h) H_2_O_2_ yield rate and corresponding FE on CoSAs/SNPs‐OCNTs at different applied potentials in H‐cell measurements. i) H_2_O_2_ yield rate and corresponding FE on CoSAs/SNPs‐OCNTs at 0.2 V (vs RHE) for 8 consecutive cycles.

The electrocatalytic 2e^−^ ORR performance of various samples was evaluated using a rotating ring‐disk electrode (RRDE) setup in O_2_‐saturated 0.1 m NaPi buffer solution at pH of 10.54. The selection of 0.1 m NaPi as the electrolyte was strategic, due to the deactivation of the Lewis acid sites in the TS‐1 catalyst under highly alkaline conditions, which impedes the subsequent ammoximation of CYC to CHO.^[^ ^9b^ ^]^ Prior to conducting the electrochemical experiments, the RRDE collection efficiency was calibrated using the ferrocyanide/ferricyanide redox reaction system, which was determined to be 0.35 (Figure S6, Supporting Information).^[^ ^20^ ^]^ Initially, we investigated the influence of catalyst loading amount on the electrocatalytic 2e^−^ ORR performance. It was discovered that the optimal catalyst loading amount was ≈0.05 mg cm^−2^, which provided a balance between ORR activity and H_2_O_2_ selectivity (Figure S7, Supporting Information). The corresponding linear sweep voltammetry (LSV) curves of the various electrocatalysts collected at 1600 rpm are exhibited in **Figure** **3**a. Obviously, CoSAs/SNPs‐OCNTs affords the most positive onset potential of 0.73 V (vs RHE) determined at the disk current density of −0.1 mA cm^−2^ and the largest H_2_O_2_ current density (*j_H2O2_*) of 2.46 mA cm^−2^ at 0.4 V (vs RHE) among these samples, speaking the fact of its highest ORR activity and two‐electron selectivity (Figure 3b). The calculated H_2_O_2_ selectivity based on LSV data also substantiates that CoSAs/SNPs‐OCNTs delivers the highest H_2_O_2_ selectivity of 95.9%, outperforming OCNTs (94.3%), CoPc (86.9%) and CoNPs‐OCNTs (57.3%) (Figure 3c). This demonstrates that the CoSAs/SNPs‐OCNTs catalyst inherits the high 2e^−^ ORR selectivity of OCNTs and boosts the ORR activity through a synergistic effect between atomically dispersed Co sites and Co NPs. Also, the electron transfer number during ORR for CoSAs/SNPs‐OCNTs was calculated to be 2.08, suggesting a nearly complete 2e^−^ ORR pathway. The ORR reaction kinetics was further analyzed by the Tafel plots derived from the kinetic currents. As depicted in Figure 3d, the CoSAs/SNPs‐OCNTs exhibits a smaller Tafel slope of 65.1 mV dec^−1^ than that of OCNTs (77.4 mV dec^−1^), CoPc (144.1 mV dec^−1^) and CoNPs‐OCNTs (88.0 mV dec^−1^), indicating its faster 2e^−^ ORR kinetics. The CoSAs/SNPs‐OCNTs catalyst also exhibits the highest mass activities at the low overpotential range among these catalysts, even comparable to that of the state‐of‐the‐art noble‐metal alloy catalysts (Figure S8, Supporting Information). To further elucidate the catalytic center, 0.1 m potassium thiocyanate (KSCN) was introduced into the electrolyte as the transition metal ion inhibitor, aimed at deactivating the metal sites.^[^ ^21^ ^]^ The results, shown in Figure 3e, demonstrate a negative shift in onset potential and a reduction in disk current density upon addition of SCN^−^, implying that Co species are the active sites for 2e^−^ ORR in CoSAs/SNPs‐OCNTs. This conclusion is in line with the low ORR activity of OCNTs as observed in Figure 3a. Additionally, the catalyst stability was investigated via performing chronoamperometric test at a constant disk and ring potential of 0.4and 1.2 V (vs RHE), respectively. Over 90% retention of disk current after 12 h underscores the high electrocatalytic stability of CoSAs/SNPs‐OCNTs (Figure 3f). The observed gradual increase in ring current and H_2_O_2_ selectivity is owing to the accumulation of low concentration of H_2_O_2_ in the electrolyte.^[^ ^22^ ^]^ Furthermore, in situ attenuated total reflectance surface‐enhanced infrared absorption spectroscopy (ATR‐SEIRAS) tests were conducted to investigate the key adsorbed oxygen intermediates on the CoSAs/SNPs‐OCNTs catalyst during ORR. The experimental set‐up for in situ ATR‐SEIRAS measurements is exhibited in Figure S9 (Supporting Information). Three peaks related to oxygen species can be detected (Figure 3g), in which the band at ≈1265 cm^−1^ is assigned to the O─O stretching vibration of adsorbed superoxide (OOH_ad_), the band at ≈1349 cm^−1^ is assigned to the OOH bending vibration of adsorbed hydroperoxide (HOOH_ad_), and the band at ≈1438 cm^−1^ is attributed to the O─O stretching vibration of weakly adsorbed molecular oxygen (O_2, ad_).^[^ ^23^ ^]^ The peak intensities of 1265 and 1349 cm^−1^ bands were proportional to the overpotentials, suggesting the ^*^OOH mediated 2e^−^ ORR pathway on CoSAs/SNPs‐OCNTs. Collectively, these results validate the superior performance of CoSAs/SNPs‐OCNTs in electrocatalytic 2e^−^ ORR, exhibiting an efficacy comparable to that of recently reported single‐atom electrocatalysts (Table S1, Supporting Information).

Given that the generated H_2_O_2_ could potentially undergo further reduction in the presence of electrocatalysts, we investigated the electrocatalytic H_2_O_2_ reduction reaction (H_2_O_2_RR) in Ar‐saturated 0.1 m NaPi solution containing 10 mM H_2_O_2_. As illustrated in Figure S10 (Supporting Information), the CoSAs/SNPs‐OCNTs catalyst exhibits a negligible H_2_O_2_ reduction current density, indicating its inertness toward H_2_O_2_RR. This characteristic suggests that CoSAs/SNPs‐OCNTs is a promising candidate for efficient H_2_O_2_ electrosynthesis. The practical H_2_O_2_ production capability of CoSAs/SNPs‐OCNTs was then evaluated in the H‐cell device by performing chronoamperometric tests at different applied potentials. The resulting current‐time (I‐t) curves are displayed in Figure S11 (Supporting Information). H_2_O_2_ yield quantification was determined based on the variation in peak absorbance after introduction of post‐ORR electrolytes into standard Ce(SO_4_)_2_ solutions, which was measured using a UV–vis spectrophotometer (Figures S12 and S13, Supporting Information).^[^ ^24^ ^]^ As exhibited in Figure 3h, the CoSAs/SNPs‐OCNTs catalyst achieves a high H_2_O_2_ yield rate of 567 ± 25 mmol g_cat_^−1^ h^−1^ with a corresponding faradaic efficiency (FE) of 88.9% ± 6.3% at 0.2 V (vs RHE) in O_2_‐saturated 0.1 M NaPi. The reusability of the catalyst is also critical in electrochemical H_2_O_2_ production. Thus, we evaluated the reusability of CoSAs/SNPs‐OCNTs by performing the consecutive cycling tests at 0.2 V (vs RHE) for 8 cycles with 2 h of each cycle (Figure S14, Supporting Information). No obvious decay of the H_2_O_2_ yield rate and FE can be detected during 8 consecutive cycles, as shown in Figure 3i and Figure S15 (Supporting Information). Moreover, the LSV curves of the initial and post‐ORR CoSAs/SNPs‐OCNTs measured in the H‐cell device also exhibit minor variation (Figure S16, Supporting Information), further suggesting the excellent reusability and electrochemical stability of CoSAs/SNPs‐OCNTs. In addition, the morphology and chemical composition of post‐ORR CoSAs/SNPs‐OCNTs were also well‐maintained (Figures S17–S19, Supporting Information), further confirming its structural stability during H_2_O_2_ electrosynthesis.

### 2.3 Theoretical Calculations and Mechanism Elucidation

**Figure 4:**
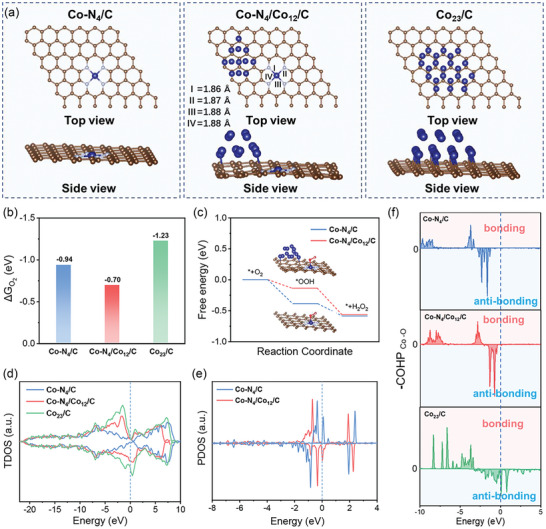
a) Optimized structural models of Co‐N_4_/C, Co‐N_4_/Co_12_/C and Co_23_/C. b) Oxygen adsorption free energy on these models. c) Gibbs free energy diagrams of 2e^−^ ORR of Co‐N_4_/C and Co‐N_4_/Co_12_/C, respectively. Inset: adsorption of ^*^OOH on Co‐N_4_/C and Co‐N_4_/Co_12_/C. Brown sphere: carbon, blue sphere: cobalt, wathet sphere: nitrogen, red sphere: oxygen, white sphere: hydrogen. d) Total density of states for Co‐N_4_/C, Co‐N_4_/Co_12_/C and Co_23_/C. e) Projected density of states on d orbitals of Co‐N_4_ for Co‐N_4_/C and Co‐N_4_/Co_12_/C. f) Projected crystal orbital Hamilton population (pCOHP) of Co from Co‐N_4_/C, Co‐N_4_/Co_12_/C and Co_23_/C models and O from adsorbed O_2_.

To explore the possible origin of the superior 2e^−^ ORR performance of CoSAs/SNPs‐OCNTs, the DFT calculations were further performed. Based on the experimental observations and reported works,^[^ ^17^, ^18^, ^19^ ^]^ the stable Co‐N_4_ configuration was established to represent the Co atomic sites. Besides, models of small Co_12_ and larger Co_23_ NPs were also developed to represent Co NPs of different sizes. Therefore, three structural models, namely Co‐N_4_/C, Co‐N_4_/Co_12_/C and Co_23_/C were constructed to elucidate the interactions between Co‐N_4_ sites and Co NPs (**Figure** **4**a). The Co─N bond length in the Co‐N_4_/C model is 1.87 Å, while in the Co‐N_4_/Co_12_/C model, the bond lengths are 1.86, 1.87, 1.88, and 1.88 Å, respectively. We first calculated the adsorption free energy of O_2_ on these structures, as the adsorption of O_2_ is the critical initial step in the ORR process (Figures S20–S22, Supporting Information). As shown in Figure 4b, the calculated adsorption free energies for O_2_ on Co‐N_4_/C, Co‐N_4_/Co_12_/C and Co_23_/C were −0.94, −0.70 and −1.23 eV, respectively. These results indicate that the Co‐N_4_/Co_12_/C model exhibits weaker adsorption of O_2_, consequently hindering its dissociation to ^*^O and ^*^OH intermediates during the ORR process leading to inhibited 4e^−^ ORR pathway. To further confirm this, the free energy diagram of the intermediate states in the 2e^−^ ORR pathway was calculated, as exhibited in Figure 4c. Since ^*^OOH is a critical intermediate in the 2e^−^ ORR, the formation free energy of the ^*^OOH intermediate (ΔG_*OOH_) is commonly regarded as a reliable descriptor for assessing the 2e^−^ ORR reactivity of a catalyst.^[^ ^25^ ^]^ Apparently, the Co‐N_4_/Co_12_/C exhibits a lower energy barrier for ^*^OOH formation (0.13 eV) compared to Co‐N_4_/C (0.38 eV), suggesting the promoted 2e^−^ ORR activity on Co‐N_4_/Co_12_/C. While, the ^*^OOH intermediate was unstable on Co_23_/C, where it easily dissociated to ^*^OH and ^*^O (Figure S23, Supporting Information), thus favoring the 4e^−^ ORR pathway. Consequently, the coexistence of Co‐N_4_ atomic sites and ultrafine Co NPs significantly boosts the activity and selectivity of the 2e^−^ ORR process.

To further investigate the underlying synergistic effects between Co SAs and Co NPs, the electronic density of states including total and projected density of states (TDOS and PDOS) of three model structures were calculated. Notably, the Co_23_/C model exhibits an increased electron occupancy near the Fermi level compared to the Co‐N_4_/C and Co‐N_4_/Co_12_/C models (Figure 4d), which promotes the electron transfer between the catalyst surface and adsorbed reaction intermediates, thereby making the Co_23_/C more conducive to the 4e^−^ ORR process.^[^ ^18^ ^]^ Conversely, the d orbitals of Co‐N_4_/Co_12_/C are moderately positioned near the Fermi level, leading to the weaker adsorption of ^*^OOH, thus the larger propensity to form H_2_O_2_. Besides, Figure 4e displays the PDOS of d band of the active Co atoms in Co‐N_4_/C and Co‐N_4_/Co_12_/C, showing a noticeable downshift of the Co 3d states to lower energy level in Co‐N_4_/Co_12_/C compared to Co‐N_4_/C. This indicates that the existence of Co_12_ effectively regulates the Co 3d orbital of Co‐N_4_ in Co‐N_4_/Co_12_/C, resulting in a favorable 2e^−^ ORR pathway. The projected crystal orbital Hamilton population (pCOHP) analysis was then conducted to quantify the energy contribution of atomic bonds,^[^ ^26^ ^]^ as shown in Figure 4f. The integration of the pCOHP up to the Fermi level (ICOHP) offers quantitative insights into the bonding and antibonding orbital populations between Co and ^*^O_2_. Typically, a more negative ICOHP value signifies a stronger bond strength between atoms.^[^ ^27^ ^]^ In this study, the Co_23_/C model possesses the most negative ICOHP value (−3.07 eV), indicative of stronger bond strength with ^*^O_2_ and consequently easier dissociation of the O─O bond. According to Sabatier's principle, the optimal adsorption energy should be neither too strong nor too weak. Therefore, the moderate ICOHP value calculated for Co‐N_4_/Co_12_/C (−2.51 eV), in comparison with Co‐N_4_/C (−2.49 eV) and Co_23_/C (−3.07 eV), suggests that Co‐N_4_/Co_12_/C achieves the most balanced adsorption process. Moreover, the introduction of Co NPs nearby Co‐N_4_ atomic sites promotes the interaction between ^*^O_2_ and the Co active sites, thereby boosting 2e^−^ ORR activity. The results from DFT calculations demonstrate that the synergistic interaction between Co SAs and Co NPs effectively tailors the electronic structure of Co‐N_4_ active sites, thus optimizing binding strength of the ^*^OOH intermediate and facilitating the 2e^−^ ORR process. These findings are consistent with the experimental results, confirming the beneficial role of the synergistic effects in enhancing electrocatalytic 2e^−^ ORR performance.^[^ ^28^ ^]^

### 2.4 Integrated Electrochemical System for CHO Production

**Figure 5:**
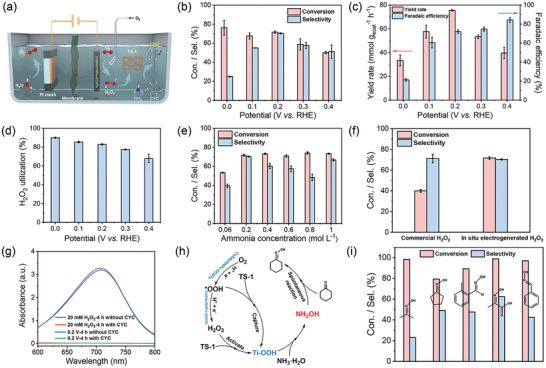
a) Schematic illustration of the integrated electrochemical system by coupling in situ electrogenerated H_2_O_2_ with the TS‐1 heterogeneous catalyst. b) CYC conversion and CHO selectivity at different applied potentials. c) CHO yield rate and corresponding FE at different applied potentials. d) H_2_O_2_ utilization efficiency in the ammoximation process at different applied potentials. e) CYC conversion and CHO selectivity at different ammonia concentrations. f) Comparison of CYC conversion and CHO selectivity between commercial H_2_O_2_ system and in situ electrogenerated H_2_O_2_ system. g) Detection of NH_2_OH in the commercial H_2_O_2_ system and in situ electrogenerated H_2_O_2_ system with or without CYC. h) The proposed mechanism of the CHO synthesis pathways in this integrated electrochemical system. i) Electrocatalytic synthesis of various oximes via this integrated electrochemical system.

The results from the RRDE and H‐cell experiments indicate that CoSAs/SNPs‐OCNTs exhibits high electrocatalytic performance for H_2_O_2_ production. Consequently, we coupled the in situ electrogenerated H_2_O_2_ on CoSAs/SNPs‐OCNTs with the TS‐1 catalyst to achieve the one‐pot synthesis of CHO under ambient conditions (**Figure** **5**a). The characterizations of TS‐1 catalyst are exhibited in Figure S24 (Supporting Information). The XRD pattern of TS‐1 shows a typical MFI structure and the SEM image displays that its particle size is ≈200 to 300 nm. The N_2_ adsorption‐desorption isotherms demonstrate that the TS‐1 possesses a high BET specific surface area of 269.6 m^2^ g^−1^ composed of microporous structure, which is conducive to the adsorption and diffusion of reactants. In addition, the band observed at 965 cm^−1^ in the Fourier transform infrared (FT‐IR) spectrum of the TS‐1 sample can be attributed to the stretching vibrations of SiO_4_ tetrahedra bound to Ti atoms, representing the Ti─O─Si bonds within the framework (Figure S25, Supporting Information).^[^ ^8^, ^29^ ^]^ Controlled potential electrolysis was subsequently conducted using CoSAs/SNPs‐OCNTs as the cathode electrocatalyst in O_2_‐saturated 0.1 m NaPi solution containing CYC, NH_3_·H_2_O, and TS‐1 (Figure S26, Supporting Information). The TS‐1 was uniformly dispersed in the electrolyte through vigorous stirring. The reaction products were analyzed using the proton nuclear magnetic resonance (^1^H NMR) (Figure S27, Supporting Information). The corresponding conversion rate of CYC and the selectivity of CHO after the reaction are manifested in Figure 5b. Notably, both the conversion rate and selectivity exhibit simultaneous enhancements at an applied potential of 0.2 V (vs RHE), where the conversion rate of CYC reaches 71.7% ± 1.1%, and the selectivity for CHO is recorded at 70.3% ± 0.6%. At higher overpotentials, the conversion rate of CYC increased due to elevated H_2_O_2_ formation; however, the selectivity toward CHO significantly decreased. This reduction in selectivity was attributed to the high concentration of H_2_O_2_ potentially oxidizing CYC to form by‐products. Conversely, at lower overpotentials, insufficient H_2_O_2_ production led to decreased conversion rates and selectivity. Therefore, 0.2 V (vs RHE) was identified as the optimal potential for this system. We then normalized the CHO yield rate by per mass unit of electrocatalyst, which can reach 75.6 ± 0.5 mmol g_ecat_^−1^ h^−1^ with a FE of 72.0% ± 1.9% at 0.2 V (vs RHE) (Figure 5c). Control experiments conducted in an electrolyte devoid of nitrogen source, O_2_, TS‐1 heterogeneous catalyst, or electricity resulted in no detectable CHO, confirming the necessity of these components for CHO synthesis (Table S2, Supporting Information). Thus, the in situ electro‐synthesized H_2_O_2_ coupled with the TS‐1 catalyst achieved efficient CHO synthesis at room temperature and atmospheric pressure. We further assessed the utilization efficiency of H_2_O_2_ by quantifying the remaining H_2_O_2_ in the post‐reaction electrolyte using the cerium sulfate titration method (Figure S28, Supporting Information). Control experiments confirm that the presence of organics and ammonia in the electrolyte does not affect the detection results (Figure S29, Supporting Information). At 0.2 V (vs RHE), the H_2_O_2_ utilization efficiency exceeded 80%, indicating the efficient activation of in situ electro‐synthesized H_2_O_2_ by TS‐1 to form Ti‐OOH active species, thereby achieving the ammoximation of CYC (Figure 5d). Subsequent control experiments adjusting the ammonia concentration revealed optimal yield and selectivity at an ammonia concentration of 0.2 m, as illustrated in Figure 5e. Further increasing the ammonia concentration resulted in declined selectivity, likely due to increased alkalinity which could deactivate the TS‐1 catalyst (Figure S30, Supporting Information).

Additionally, we performed a control experiment by directly adding commercial H_2_O_2_ with the similar concentration as generated by the CoSAs/SNPs‐OCNTs catalyst to the ammoxiamtion process. As depicted in Figure S31 (Supporting Information) and Figure 5f, the oxime production in the commercial H_2_O_2_ system was significantly lower than that observed with in situ electrogenerated H_2_O_2_ system under the identical reaction conditions, thus clarifying the superiority of in situ H_2_O_2_ generation. This phenomenon demonstrates that the reaction intermediates or free radicals formed during the 2e^−^ ORR process played a critical role in enhancing the activity of the reaction. Therefore, we further carried out the electron paramagnetic resonance (EPR) experiments by using 5,5‐dimethyl‐1‐pyrroline N‐oxide (DMPO) as the free radical trapping agent to identify the significant role of free radicals in the system. As exhibited in Figure S32 (Supporting Information), the decomposition of H_2_O_2_ in the commercial H_2_O_2_ system primarily generated •OH radicals, which were captured by DMPO. Nevertheless, in the in situ electrogenerated H_2_O_2_ system, both •OH and •OOH radical signals were observed, indicating that the ^*^OOH intermediate produced on CoSAs/SNPs‐OCNTs can be transferred to the electrolyte by DMPO capturing. Moreover, when coupled with the TS‐1 catalyst, it can also act as the trapping agent to bind with the ^*^OOH intermediate generated during the 2e^−^ ORR process to form Ti‐OOH active species, which subsequently oxidized CYC to CHO with enhanced catalytic activity.^[^ ^9^, ^29^ ^]^ To further confirm this, in situ Raman spectroscopy was employed to directly detect the intermediates formed on the catalyst surface during the reaction. The experimental set‐up for in situ Raman measurements is exhibited in Figure S33a (Supporting Information). As the applied potential decreased, the characteristic peaks corresponding to ^*^OOH at ≈791 cm^−1^ and Ti‐OOH at ≈829 cm^−1^ were detected (Figure S33b, Supporting Information). Below 0.3 V (vs RHE), these peaks exhibited diminished intensity, likely due to the accelerated consumption of ^*^OOH associated with enhanced reaction kinetics, consistent with observations reported in the literature.^[^ ^29^ ^]^ Therefore, the in situ electrogenerated H_2_O_2_ system effectively circumvented the adsorption and activation steps of H_2_O_2_ on TS‐1 resulting in reduced energy consumption and fast reaction kinetics. This integrated electrochemical strategy, which involved coupling in situ electrogenerated H_2_O_2_ with the TS‐1 catalyst, demonstrated enhanced performance in the production of CHO, surpassing other electrochemical reduction coupling reactions (Table S3, Supporting Information).

Typically, the ammoximation reaction proceeds through the hydroxylamine pathway with NH_2_OH serving as the N‐containing active species. Control experiments also confirmed that CHO was formed immediately upon mixing NH_2_OH and CYC at room temperature, even without the application of electrical input, indicating that the coupling reaction between NH_2_OH and CYC occurred spontaneously (Entry 6 in Table S2, Supporting Information). To determine whether NH_2_OH was generated during the reaction, we conducted additional control experiments and used 8‐hydroxyquinoline as a colorimetric reagent to detect NH_2_OH via UV–vis spectroscopy (Figure S34, Supporting Information).^[^ ^30^ ^]^ The experimental results indicated that NH_2_OH was generated in the electrolyte in the absence of CYC, with a concentration similar to that produced by the exogenously added H_2_O_2_ (Figure 5g). Conversely, upon the addition of CYC to the electrolyte, no NH_2_OH was detected, implying its rapid consumption during the C─N coupling reaction with CYC. Therefore, in light of literature reports and experimental evidence, the proposed reaction mechanism is illustrated in Figure 5h: 1 O_2_ was initially electrocatalytically reduced to H_2_O_2_ on CoSAs/SNPs‐OCNTs catalyst with ^*^OOH as the reaction intermediate; 2 the in situ electrogenerated ^*^OOH intermediate and H_2_O_2_ present in the electrolyte were captured and activated by TS‐1 catalyst, forming Ti‐OOH active species; 3 Ti‐OOH effectively catalyzed the oxidation reaction of NH_3_ to NH_2_OH; 4 the generated NH_2_OH rapidly reacted with CYC to form CHO through a nucleophilic addition‐elimination process. We also conducted substrate expansion experiments using acetone, cyclopentanone, 2‐methylbenzaldehyde, pyruvic acid, and benzaldehyde as substrates to investigate the universality of the strategy (Figures S35–S39, Supporting Information). It was found that all of the selected chemicals can be converted to the corresponding oximes (Figure 5i), indicating that the strategy developed in this work has a broad applicability and can be extended to different substrates.

## 3 Conclusions

In summary, we present an integrated electrochemical approach for the sustainable synthesis of CHO by coupling in situ electrogenerated H_2_O_2_ with the TS‐1 catalyst. In this system, the CoSAs/SNPs‐OCNTs, composed of atomically dispersed Co sites and ultrafine Co NPs, served as the 2e^−^ ORR electrocatalyst and delivered a high H_2_O_2_ yield rate for the subsequent ammoximation reaction. The excellent electrocatalytic 2e^−^ ORR performance was attributed to the synergistic interaction between Co‐N_4_ atomic sites and Co NPs, where the introduction of Co NPs significantly modulated the electronic structure of Co‐N_4_ active sites resulting in the optimal binding strength of the ^*^OOH intermediate, as uncovered by the DFT calculations. When coupling the CoSAs/SNPs‐OCNTs electrocatalyst with the TS‐1 catalyst, the proposed system exhibited outstanding performance for CHO synthesis, achieving a high CYC conversion rate of 71.7% ± 1.1%, CHO selectivity of 70.3% ± 0.6%, CHO yield rate of 75.6 ± 0.5 mmol g_ecat_^−1^ h^−1^ with the FE of 72.0% ± 1.9% at 0.2 V (vs RHE). Control experiments combined with EPR characterization demonstrated that the in situ electrogenerated ^*^OOH intermediate and H_2_O_2_ present in the electrolyte can be captured and activated by the TS‐1 catalyst, forming Ti‐OOH active species. Such species promoted the formation of NH_2_OH, a crucial N‐containing active species, which subsequently reacted with CYC to produce CHO through a spontaneous process. In addition, this strategy can be extended to the synthesis of other oximes, manifesting its broad applicability across various substrates. This work provides a promising approach for C─N bond formation in oximes and facilitates organic synthesis through the coupling of in situ electrogenerated H_2_O_2_.

## Conflict of Interest

The authors declare no conflict of interest.

## Supporting information



Supporting Information

## Data Availability

The data that support the findings of this study are available in the supplementary material of this article.
