# Supplementary material for: Ambient Synthesis of Cyclohexanone Oxime via In Situ Produced Hydrogen Peroxide over Cobalt‐Based Electrocatalyst
Source: Adv Sci (Weinh). 2024 Dec 17;12(6):2413475. doi: 10.1002/advs.202413475 (PMC11809397; doi:10.1002/advs.202413475)
Supplement: Supplementary file 1 — Supporting Information [file ADVS-12-2413475-s001.docx]

Supporting Information

Ambient Synthesis of Cyclohexanone Oxime *via* *in situ* Produced Hydrogen Peroxide over Cobalt-Based Electrocatalyst

*Hui Xu,^‡^ Meng Jin,^‡^ Shengbo Zhang,^‡^ Xinyuan Zhang, Min Xu, Yunxia Zhang, Guozhong Wang and Haimin Zhang^*^*

H. Xu, M. Jin, S. Zhang, X. Zhang, M. Xu, Y. Zhang, G. Wang, H. Zhang

Key Laboratory of Materials Physics, Centre for Environmental and Energy Nanomaterials, Anhui Key Laboratory of Nanomaterials and Nanotechnology, Institute of Solid State Physics, HFIPS, Chinese Academy of Sciences, Hefei 230031, China.

E-mail: zhanghm@issp.ac.cn

H. Xu, M. Jin, S. Zhang, X. Zhang, M. Xu, Y. Zhang, G. Wang, H. Zhang

University of Science and Technology of China, Hefei 230031, China.

** Corresponding Author.*

*‡**These authors contributed equally to this work.*

Table of Contents

1. Experimental Section: Pages 3-6

**2. Figures and Tables: Pages 7-31**

1. Experimental Section

**Materials and reagents**

Carboxylic multi-walled carbon nanotubes (OCNTs), cobalt phthalocyanine (CoPc), melamine (C_3_H_6_N_6_), sodium carbonate (Na_2_CO_3_), potassium ferricyanide (K_3_[Fe(CN)_6_]), ceric sulfate (Ce(SO_4_)_2_), potassium thiocyanate (KSCN), titanium silicate-1 (TS-1), cyclohexanone (C_6_H_10_O), d_6_-DMSO, maleic acid (C_4_H_4_O_4_) and 8-hydroxyquinoline (C_9_H_7_NO) were obtained from Aladdin Chemical Reagent Co., Ltd. Sodium hydrogen phosphate (Na_2_HPO_4_), sodium dihydrogen phosphate dihydrate (NaH_2_PO_4_·2H_2_O), sulfuric acid (H_2_SO_4_), hydrogen peroxide (H_2_O_2_) and aqueous ammonia (NH_3_·H_2_O) were purchased from Sinopharm Chemical Reagent Co., Ltd. Nafion solution (5 wt.%) was obtained from Shanghai Hesen electric Co., Ltd. Graphite felt (GF) was supplied by Beijing Jinglong Special Carbon Technology Co., Ltd. All chemical reagents were utilized as received in the synthesis without any additional treatment.

**Synthesis of CoSAs/SNPs-OCNTs**

Firstly, 0.1 g of OCNTs and 0.5 g of melamine were added into 100 mL of a mixture solvent of ethanol and deionized water with a volume ratio of 1:1 for sonicating 1 h. Then 10 mg of CoPc was dissolved in 5 mL of ethanol, followed by dropwise adding into the above mixture solvent under vigorous stirring. The assembly process of CoPc and the OCNTs support was conducted for 24 h under continuous stirring, subsequently the precipitate was separated, washed by ethanol and dried at 60 ℃. The obtained product was annealed at 900 ℃ for 1 h with a ramping rate of 5 ℃/min under Ar atmosphere, followed by natural cooling down to room temperature. Finally, the resultant product was immersed in 4 M H_2_SO_4_ at 120 ℃ for 6 h to remove the Co-related large nanoparticles, then cleaned by water and ethanol, and dried for further use.

**Synthesis of CoNPs-OCNTs**

The CoNPs-OCNTs sample was fabricated in a similar approach but without the acid-etching process.

**Characterization**

The crystalline structures of samples were identified by the X-ray diffraction analysis (XRD, Philips X’pert PRO) using Nifiltered monochromatic Cu Kα radiation (λKα1=1.5418 Å) at 40 kV and 40 mA. Scanning electron microscope (SEM) images of samples were obtained using SU8020 (Hitachi, Japan) with an accelerating voltage of 10.0 kV. Transmission electron microscope (TEM) images of samples were obtained using JEMARM 200F operating at an accelerating voltage of 200 kV. Raman spectrum was measured on a XploRA PLUS Raman spectrometer (Horiba Scientific) using 532 nm laser excitation. Nitrogen adsorption-desorption isotherms were measured using an automated gas sorption analyzer (Autosorb-iQ-Cx). The Co contents were determined by the inductively coupled plasma atomic emission spectrometer (ICP-AES, ICP-6300, Thermo Fisher Scientific). X-ray photoelectron spectroscopy (XPS) analyses of the samples were performed on an ESCALAB 250 X-ray photoelectron spectrometer (Thermo, America) equipped with Al Kα1, 2 monochromatized radiations at 1486.6 eV X-ray source. The synchrotron-based X-ray absorption measurements were performed at the 1W1B station of Beijing Synchrotron Radiation Facility, China. Absorbance data were obtained on SHIMADZU UV-2700 Ultraviolet-visible (UV-vis) spectrophotometer. Fourier-transform infrared spectroscopy (FT-IR) spectrum of TS-1 was measured on a Nicolet-Nexus spectrometer (Thermo Nicolet Corporation) by the KBr pellet technique. Electron paramagnetic resonance (EPR, Bruker EMX plus, Germany) was used to detect the free radicals produced during the electrolysis process with 5,5-dimethyl-1-pyrroline-N-oxide (DMPO) as the spin trapping agent.

**Electrochemical measurements**

The ORR catalytic performances were measured on a CHI 760E electrochemical workstation (CH Instrumental Corporation, Shanghai, China) with a three-electrode configuration and a rotating ring-disk electrode setup (RRDE, Pine Instruments Corporation) under ambient conditions. A RRDE electrode with a glassy carbon electrode (0.247 cm^2^ area) and a platinum ring electrode (0.186 cm^2^ area) was used as the working electrode. Prior to the measurements, the electrode was polished with 0.3 and 0.05 μm alumina suspensions on a polishing cloth and rinsed with deionized water for several times. The catalyst ink was prepared by dispersing 2.5 mg as-synthesized catalysts into 980 μL of ethanol and 20 μL of Nafion solution (5.0 wt.%), followed by ultrasonication for 30 min to get homogeneous ink. Then 5 μL of catalyst ink was drop-casted onto the disk electrode and naturally dried. The catalyst loading amount was 0.05 mg cm^-2^. A Hg/HgO electrode and a Pt wire was used as the reference and counter electrode, respectively. 0.1 M NaPi (adjusted pH to 10.54 by 0.5 M Na_2_CO_3_) was used as the electrolyte in this work. All potentials of the electrochemical measurements were converted into reversible hydrogen electrode (RHE) by using the following equations:

*E_RHE_ = E_Hg/HgO_+0.059×pH+0.098*

Before the measurements, all catalysts were electrochemical activated by performing cyclic voltammetry (CV) at 100 mV s^-1^ and 1600 rpm until stable voltammogram curves were acquired. The linear sweep voltammetry (LSV) curves were recorded in O_2_-saturated condition with a scan rate of 10 mV s^-1^ at 1600 rpm and the potential of the Pt ring electrode was set at 1.2 V (*vs.* RHE) to detect as-generated H_2_O_2_ on disk electrode. The H_2_O_2_ selectivity and electron transfer number (*n*) on the RRDE were calculated using the following equations:

*Selectivity (%) = 200 × (I_r_/N_c_)/(I_d_+I_r_/N_c_)*

*n = 4×I_d_/(I_d_+I_r_/N_c_)*

where *I_r_* and *I_d_* are the ring current and the disk current, respectively, and *N_c_* is the collection efficiency of the ring electrode (0.35 after calibration by [Fe(CN)_6_]^3-^/^4-^ redox system). We note that, for the ease of directly evaluating H_2_O_2_ current density (*j*_H2O2_), the ring current density is further adjusted by collection efficiency:

*j_H2O2_ = I_r_/(A_r_×N_c_)*

where *A_r_* is the geometric area of the ring electrode. The kinetic current (*I_k_*) was further extracted by correcting the mass transport losses, according to the K-L equations:

*1/I = 1/I_l_+1/I_k_*

*I_l_ = 0.62nFAD_O2_^2/3^ω^1/2^ν^-1/6^C_O2_*

where *I*, *I_l_* and *I_k_* are the measured total current, diffusion-limited current and kinetic current, respectively, *n* is the total electron transfer number obtained from RRDE tests, *F* is the faraday constant (96485 C mol^-1^), *A* is the geometric area of the disk electrode (0.247 cm^2^), *D_O2_* is the diffusion coefficient of O_2_ in the electrolyte (1.9×10^-5^ cm^2^ s^-1^), ω is the angular velocity of the electrode (rad s^-1^), *ν* is the kinematic viscosity of the electrolyte (0.01 cm^2^ s^-1^) and *C_O2_* is the concentration of O_2_ (1.2×10^-6^ mol cm^-3^).

***In situ* ATR-SERIES measurements**

The *in situ* attenuated total reflection surface enhanced infrared adsorption spectroscopy (ATR-SEIRAS) analyses were conducted using a FT-IR spectrometer (Nicolet iS50, Thermo Scientific) equipped with an MCT-A detector and a silicon prismatic window. Initially, CoSAs/SNPs-OCNTs ink was carefully drop-casted onto a gold film, which had been chemically deposited on the surface of the silicon prism prior to each experiment. This silicon prism with the deposited gold film and catalyst served as the working electrode. A Pt wire and a Hg/HgO electrode were used as the counter and reference electrodes, respectively. The O_2_-saturated 0.1 M NaPi solution was employed as the electrolyte. Infrared absorption spectra were recorded by averaging 128 scans at a resolution of 4.0 cm^-1^. Prior to each set of measurements, a background spectrum of the catalyst electrode was obtained at open-circuit voltage. The electrocatalytic measurements were conducted over a potential range from 0.7 to -0.3 V (*vs.* RHE) with potential increments of 0.1 V.

**Quantification of generated H_2_O_2_**

The practical yield of H_2_O_2_ was conducted in a two-compartment three-electrode H-cell device separated by a Nafion 211 membrane. Each compartment was filled with 30 mL of 0.1 M NaPi and the cathode was purged with O_2_ for at least 30 min to reach saturation. The working electrode was fabricated by drop-casting the CoSAs/SNPs-OCNTs ink onto a 1.0 × 1.0 cm^2^ commercial graphitic felt (GF) with a catalyst loading amount of 0.2 mg cm^-2^. A Pt mesh and a Hg/HgO electrode were used as the counter and reference electrodes, respectively. Then, bulk ORR electrolysis was conducted at various applied potentials for 2 h, subsequently the H_2_O_2_ yield operated at different potentials was quantified according to the ceric sulfate titration method using the following reaction:

2Ce^4+^ + H_2_O_2_ → 2Ce^3+^ + 2H^+^ + O_2_

where yellow-colored Ce^4+^ can be reduced by H_2_O_2_ to colorless Ce^3+^, thus the yield of H_2_O_2_ can be calculated by the consumption of Ce^4+^ using the following equation:

*C_H2O2_=[V_Ce4+_×C_Ce4+before_-(V_Ce4+_+V_electrolyte_)×C_Ce4+after_]/(2×V_electrolyte_)*

where V_Ce4+_ is the volume of added Ce(SO_4_)_2_, C_Ce4+before_ and C_Ce4+after_ are the concentration of Ce^4+^ before and after reaction, respectively, V_electrolyte_ is the volume of injected electrolyte after reaction. The standard concentration-absorbance curve was obtained by linearly fitting the absorbance values at 319 nm for various known concentrations of Ce^4+^. The faradaic efficiency (FE) value was calculated using the following equation:

*FE (%) = (2×C×V×F)/Q×100%*

where F is the Faraday constant (96485 C mol^-1^); C is the concentration of generated H_2_O_2_; V is the volume of electrolyte (30 mL); and Q is the passed charge during the electrolysis.

**Electrochemical synthesis of cyclohexanone oxime**

The ambient synthesis of cyclohexanone oxime (CHO) *via* *in situ* electro-synthesized hydrogen peroxide was also conducted in a two-compartment three-electrode H-cell device. The cathode compartment was filled with 30 mL of 0.1 M NaPi containing 10 mM cyclohexanone (CYC), 0.2 M NH_3_·H_2_O and 120 mg TS-1. Before tests, the cathode compartment was purged with O_2_ for at least 30 min to reach saturation. Commercial GF with a catalyst loading amount of 0.5 mg cm^-2^ (1.0 × 1.0 cm^2^) was used as the working electrode. A Pt mesh and a Hg/HgO electrode were used as the counter and reference electrodes, respectively. The electrochemical measurements were performed at different applied potentials for 4 h, then the products after reaction were further analyzed by ^1^H NMR spectroscopy. The quantification of CYC and CHO was based on the selected ^1^H NMR peak integration ratios relative to the maleic acid internal standard. The CYC conversion and CHO selectivity were calculated as follows:

*CYC conversion (%) = [n_i_(CYC)–n_f_(CYC)]/n_i_(CYC)×100%*

*CHO selectivity (%) = n(CHO)/[n_i_(CYC)–n_f_(CYC)]×100%*

where *n_i_(CYC)* and *n_f_(CYC)* are the initial and final amount (mmol) of CYC, *n(CHO)* is the generated amount of CHO, respectively.

**Detection of hydroxylamine**

Hydroxylamine (NH_2_OH) in the electrolyte was determined by the colorimetric method. First, 1 mL of electrolyte after reaction was took and diluted with 3 mL of 0.05 M phosphate buffer solution (pH=6.8). Then 1 mL of 1 wt% 8-hydroxyquinoline and 1 mL of 1 M Na_2_CO_3_ solution were injected into the above solution. Finally, under dark conditions, the solution in the water bath was heated at 100 ° C for 1 min, and then the absorbance at 708 nm was measured by UV-vis spectrophotometer.

**Theoretical calculations**

The spin polarized DFT calculations were performed using the Vienna Ab initio Simulation Package (VASP).^[1]^ The projector augmented wave (PAW) method has been used to describe the ion-electron interaction.^[2]^ The electronic exchange-correlation effects were described with Perdew-Burke-Ernzerhof generalized gradient approximation (PBE-GGA) functional.^[3]^ A cut off energy of 450 eV was used for the expansion of the wave functions. The convergence threshold was set as 10^-5^ eV in energy and 0.02 eV Å^-1^ in force. To evaluate the ORR performance of Co single atom and Co nanoparticle, the slab models of Co-N_4_/C, Co-N_4_/Co_12_/C and Co_23_/C were built. The Brillouin zone was sampled with a 2 × 1 × 1 Monkhorst-Pack k-points grid for geometric optimization of slab models, respectively. The vacuum distance was set to 15 Å to minimize the artificial interactions of the interlayer. During the total calculations, the symmetry was switched off and the dipolar correction was also included. DFT-D3 method was used for the dispersion correction.^[4]^ The change of the Gibbs free energy of each step was calculated as followings equations:

*∆G = ∆E ＋ ∆E_ZPE_ - T∆S*

where ∆E is the energy change between the reactant and product species, ∆E_ZPE_ is the difference in zero-point energy, T is the temperature (T = 298.15 K) and ∆S is the entropy difference.

The crystal orbital Hamilton population (COHP) analysis was performed using Local Orbital Basis Suite Toward Electronic Structure Reconstruction (LOBSTER) package.^[5]^

**2. Figures and Tables**


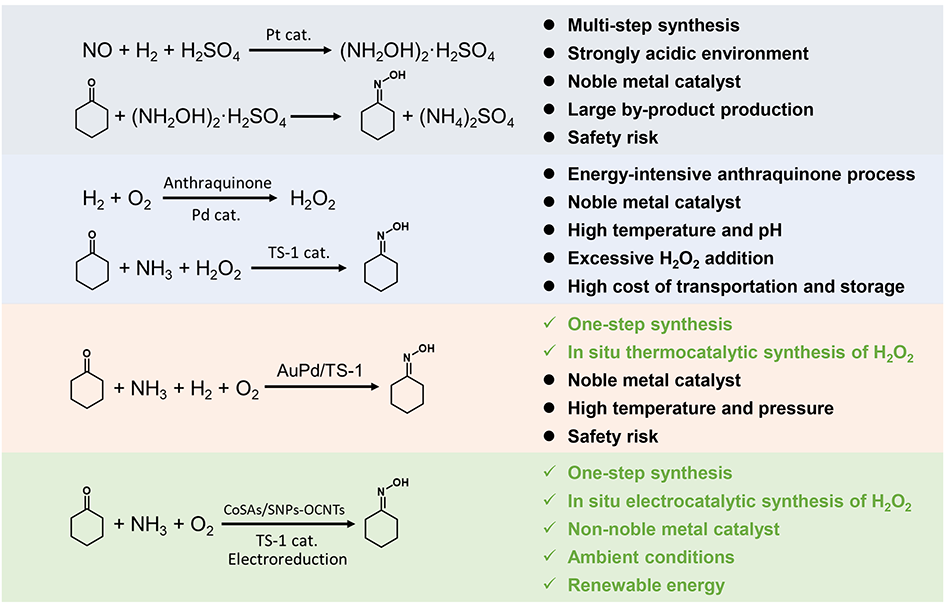


**a**

**b**

**c**

**d**

**Figure S1.** Schematic comparisons of CHO generation routes. (a) CYC-hydroxylamine method, (b) CYC ammoximation method, (c) recently reported CYC ammoximation by *in situ* produced H_2_O_2_ from H_2_ and O_2_ and (d) the proposed approach by coupling *in situ* electrogenerated H_2_O_2_ with ammoximation reaction in this work.


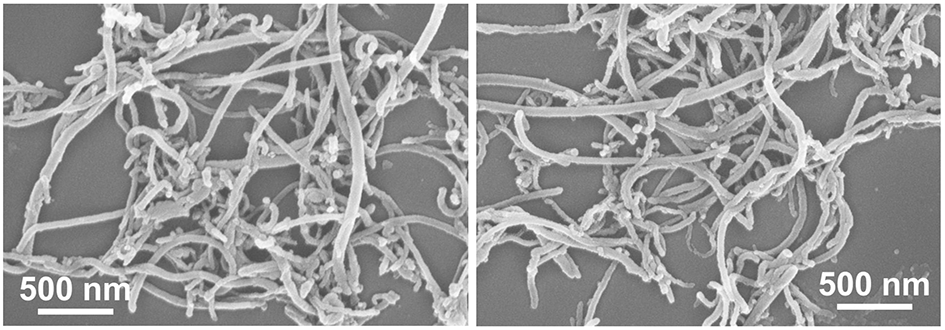


**a**

**b**

**Figure S2.** SEM images of (a) CoNPs-OCNTs and (b) CoSAs/SNPs-OCNTs.


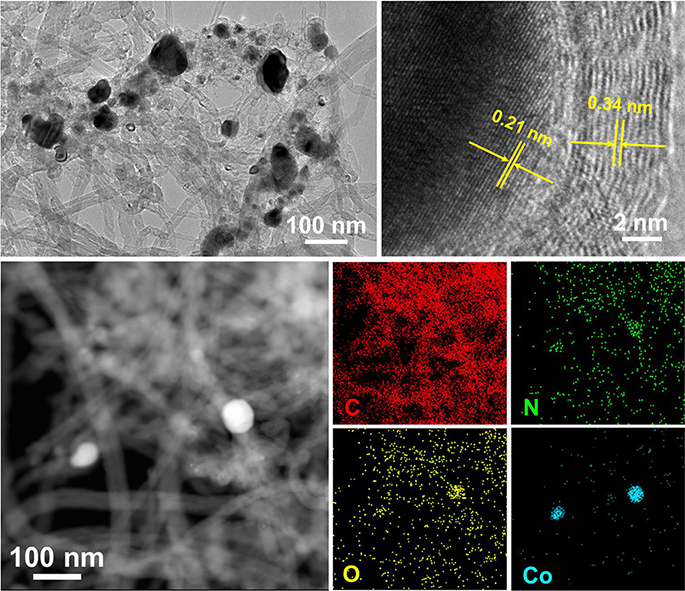


**a**

**b**

**c**

**Figure S3.** (a) TEM, (b) HRTEM, (c) HAADF-STEM images and corresponding elemental mapping images of CoNPs-OCNTs.


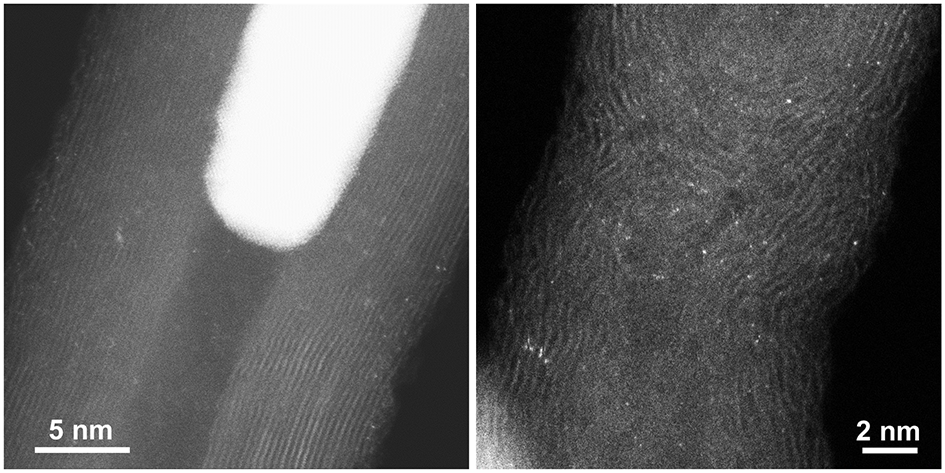


**a**

**b**

**Figure S4.** (a-b) Aberration-corrected high-angle annular dark-field scanning transmission electron microscopy (HAADF-STEM) images of CoSAs/SNPs-OCNTs at different locations.


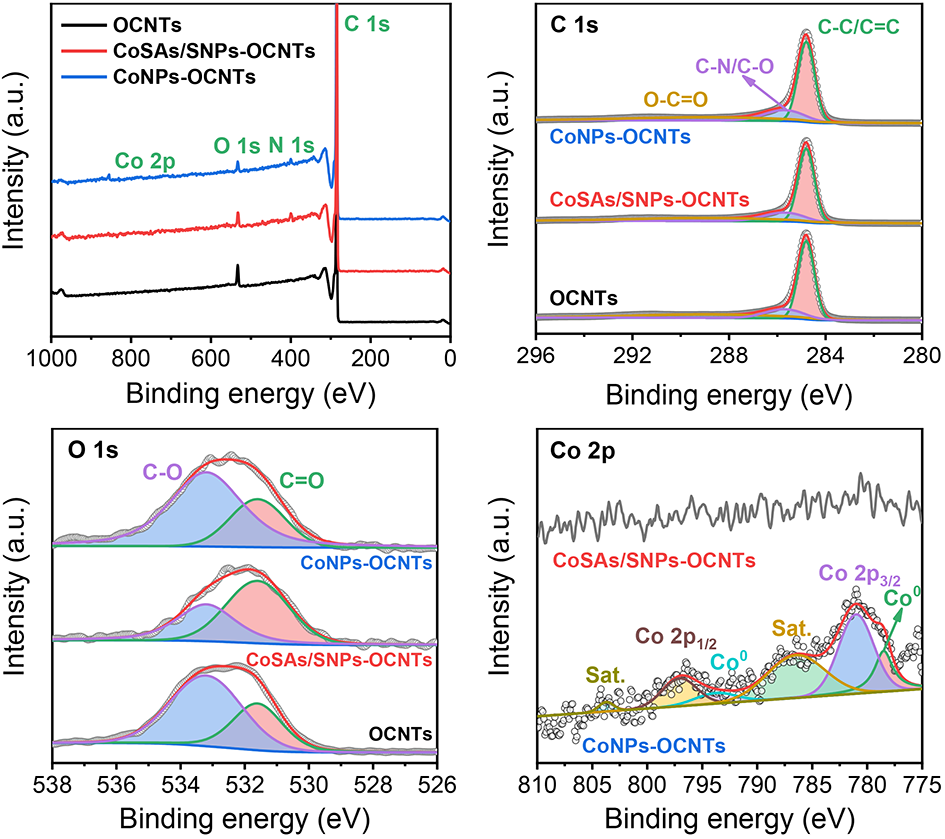


**a**

**c**

**b**

**d**

**Figure S5.** (a) XPS survey spectrum of OCNTs, CoSAs/SNPs-OCNTs and CoNPs-OCNTs. High-resolution (b) C 1s and (c) O 1s XPS spectra of OCNTs, CoSAs/SNPs-OCNTs and CoNPs-OCNTs. (d) High-resolution Co 2p XPS spectra of CoSAs/SNPs-OCNTs and CoNPs-OCNTs.


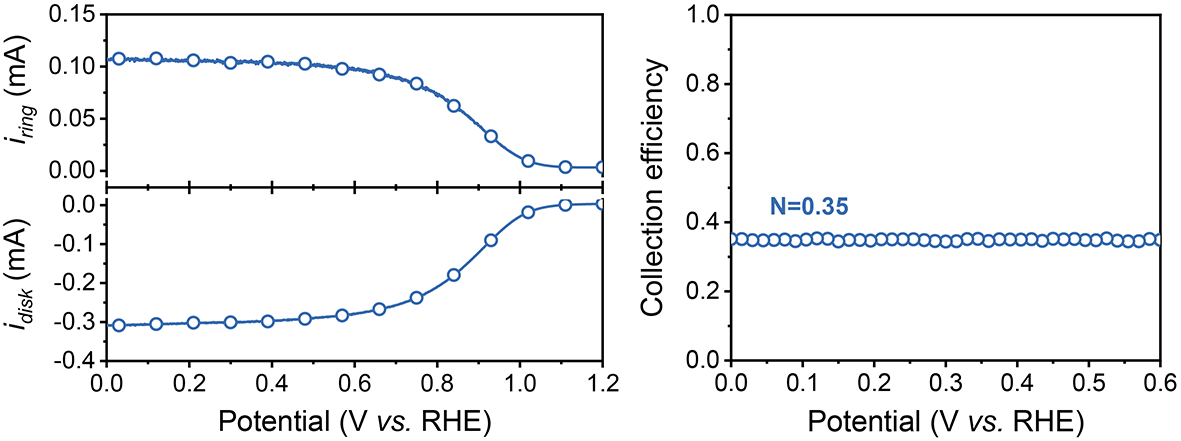


**a**

**b**

**Figure S6.** (a) LSV curves of the bare RRDE measured in Ar-saturated 0.1 M NaPi containing 2.0 mM K_3_[Fe(CN)_6_] collected at 1600 rpm. (b) Calibrated collection efficiency (N) based on LSV data *via* dividing the ring current by the disk current. The collection efficiency (N) was calibrated to be 0.35, which is close to the theoretical value (0.37).


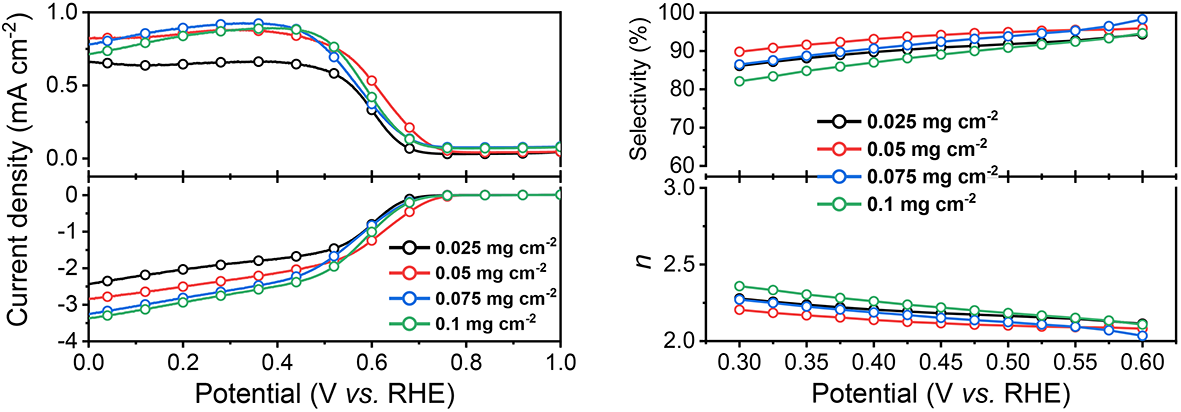


**a**

**b**

**Figure S7.** (a) LSV curves of CoSAs/SNPs-OCNTs with different loading amount obtained at 1600 rpm under 10 mV s^-1^ in O_2_-saturated 0.1 M NaPi. (b) Calculated H_2_O_2_ selectivity and electron transfer number (*n*) during ORR based on the LSV data.

It was observed that increasing the loading amount of CoSAs/SNPs-OCNTs enhanced the ORR activity and H_2_O_2_ selectivity. However, a further increase in the loading to 0.1 mg cm^-2^ resulted in a decline in performance, which can be attributed to the formation of a thicker catalyst layer. This layer potentially obscured the active sites and extended the diffusion pathways. Consequently, the generated H_2_O_2_ may be trapped within the catalyst layer, leading to its further reduction and a decrease in H_2_O_2_ selectivity. Therefore, the loading amount of the catalyst emerges as a critical parameter in the 2e^-^ ORR process. The optimal catalyst loading for CoSAs/SNPs-OCNTs was determined to be 0.05 mg cm^-2^, which effectively balances the catalytic activity and H_2_O_2_ selectivity.

**
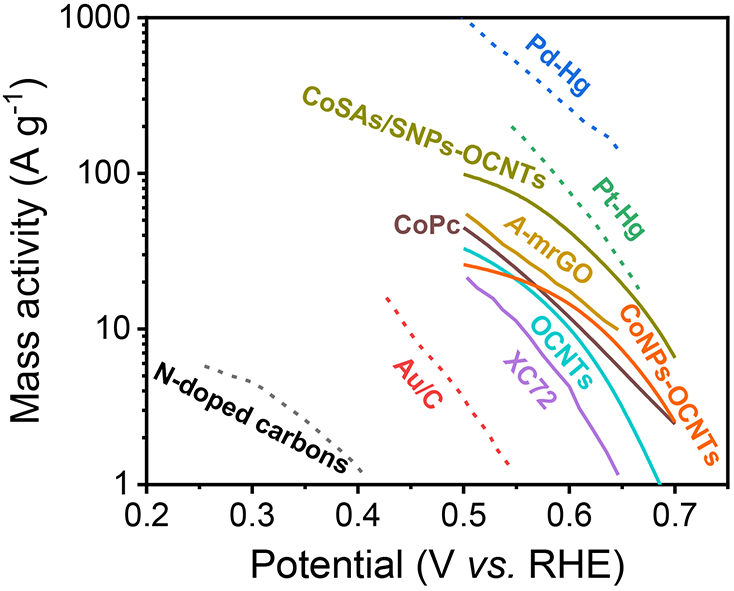
**

**Figure S8.** Mass activity of different electrocatalysts for H_2_O_2_ production. The data presented as dashed lines were measured in acidic conditions (0.1 M HClO_4_) and the data presented as solid lines were measured in basic conditions (0.1 M KOH or 0.1 M NaPi). Data were taken from precious metals and their alloys^[6]^ and carbon-based catalysts^[7]^. The mass activity was calculated by kinetic currents normalized by the catalyst mass.

**
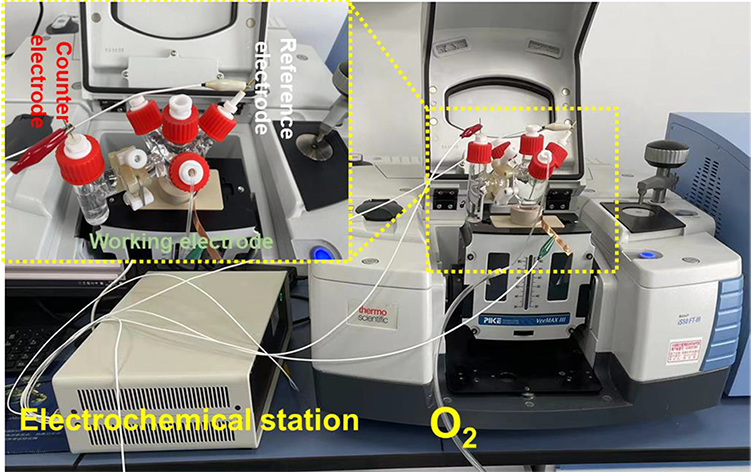
**

**Figure S9.** The optical photograph of *in situ* ATR-SEIRAS set-up.

**
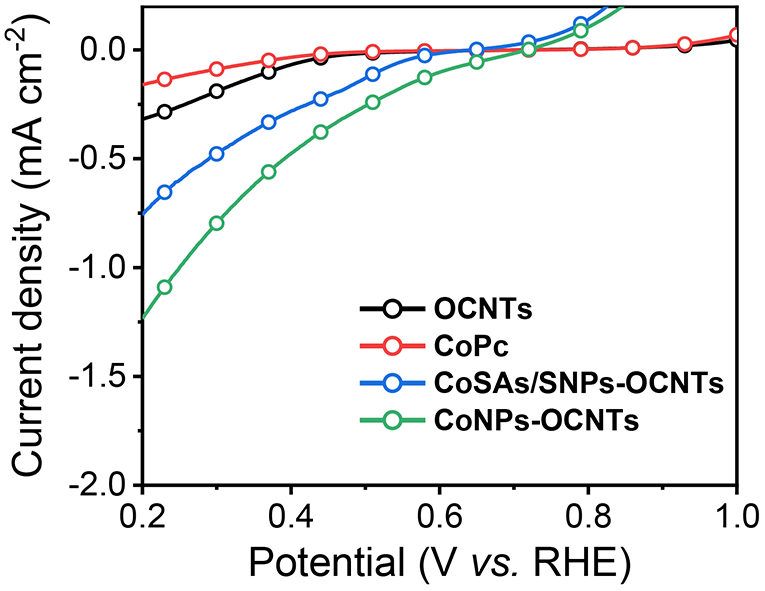
**

**Figure S10.** H_2_O_2_ reduction reaction tests for various catalysts in Ar-saturated 0.1 M NaPi containing 10 mM H_2_O_2_.

**
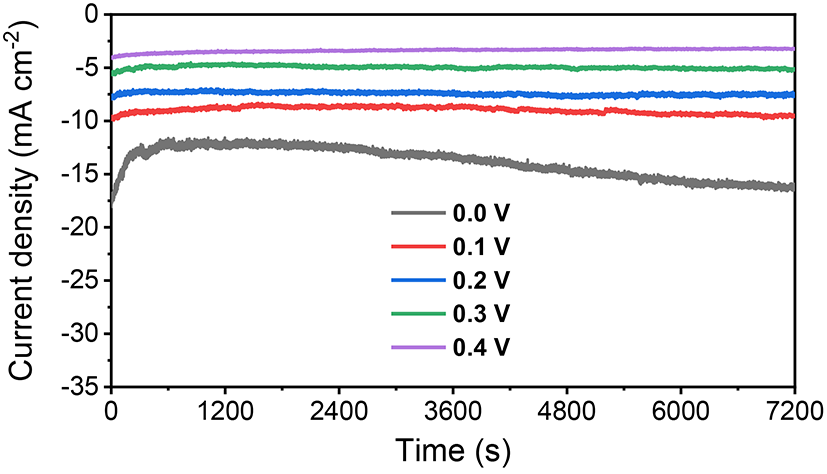
**

**Figure S11.** Chronoamperometric curves of CoSAs/SNPs-OCNTs operated at various applied potentials in O_2_-saturated 0.1 M NaPi for 2 h.


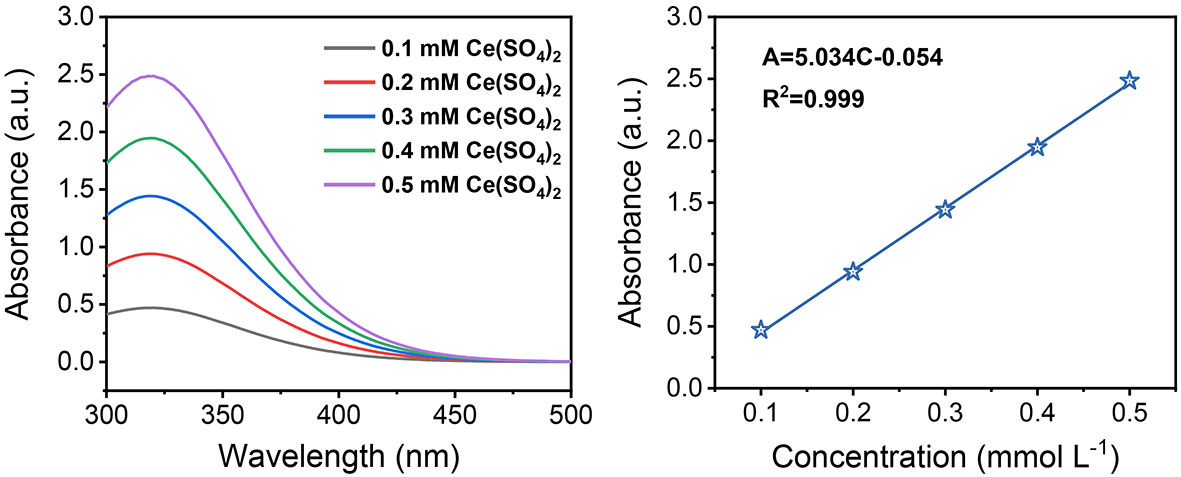


**a**

**b**

**Figure S12.** (a) Ultraviolet-visible (UV-vis) absorption spectra of standard Ce(SO_4_)_2_ solutions with different concentrations. (b) Linear peak absorbance-concentration calibration curve at 319 nm.


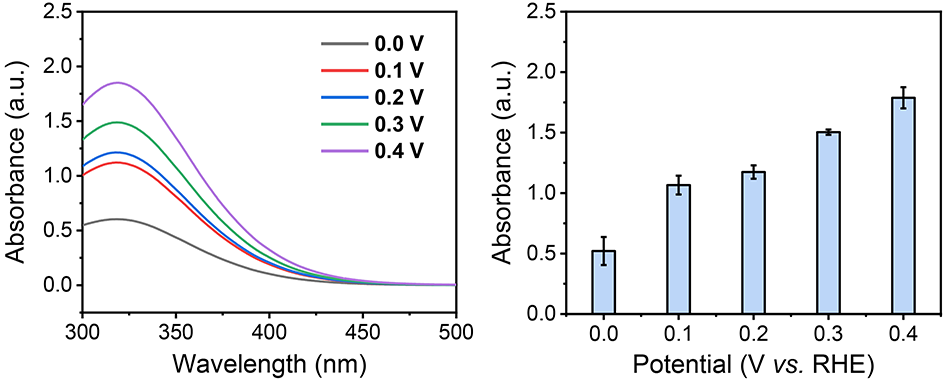


**a**

**b**

**Figure S13.** (a) UV-vis absorption spectra of Ce(SO_4_)_2_ solutions after injecting electrolytes at different electrolysis potentials over CoSAs/SNPs-OCNTs. To calculate the yield of H_2_O_2_, we injected 50 μL of electrolytes into 3.0 mL of 0.5 mM Ce(SO_4_)_2_ solutions. (b) Absorbance values at 319 nm of Ce(SO_4_)_2_ solutions after injecting electrolytes at different electrolysis potentials.

**
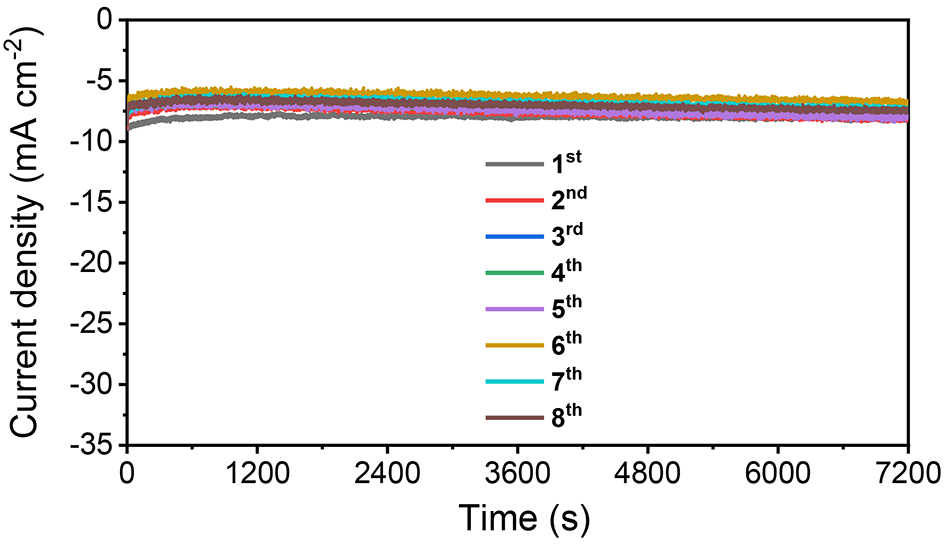
**

**Figure S14.** Chronoamperometric curves of CoSAs/SNPs-OCNTs operated at 0.2 V (*vs.* RHE) in O_2_-saturated 0.1 M NaPi for 8 consecutive cycles.


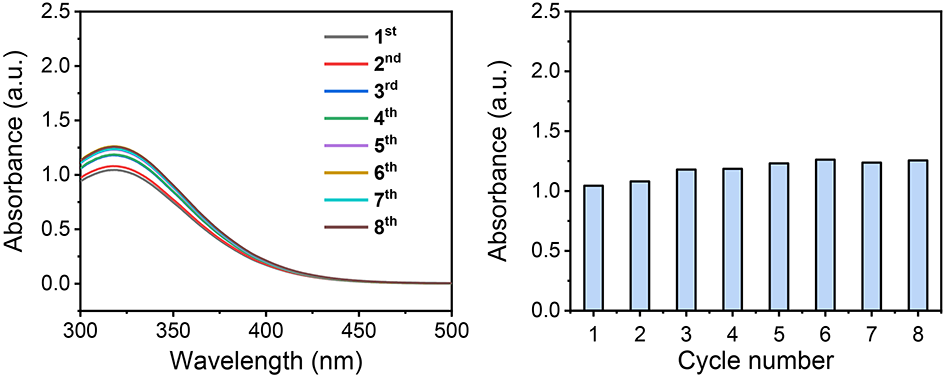


**a**

**b**

**Figure S15.** (a) UV-vis absorption spectra of Ce(SO_4_)_2_ solutions after injecting electrolytes operated at 0.2 V (*vs.* RHE) of different cycles over CoSAs/SNPs-OCNTs. To calculate the yield of H_2_O_2_, we injected 50 μL of electrolytes into 3.0 mL of 0.5 mM Ce(SO_4_)_2_ solutions. (b) Absorbance values at 319 nm of Ce(SO_4_)_2_ solutions after injecting electrolytes operated at 0.2 V (*vs.* RHE) of different cycles.

**
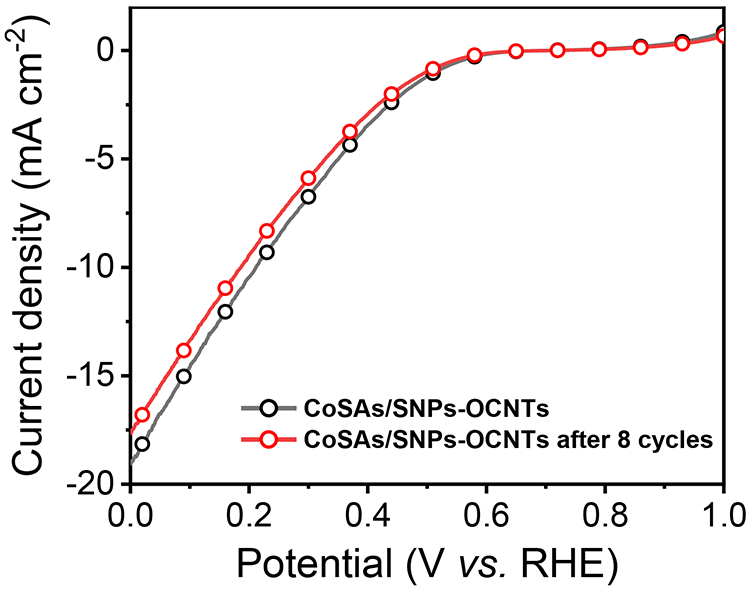
**

**Figure S16.** Comparison of LSV curves of initial CoSAs/SNPs-OCNTs and spent CoSAs/SNPs-OCNTs after 8 cycles measured in the H-cell device.


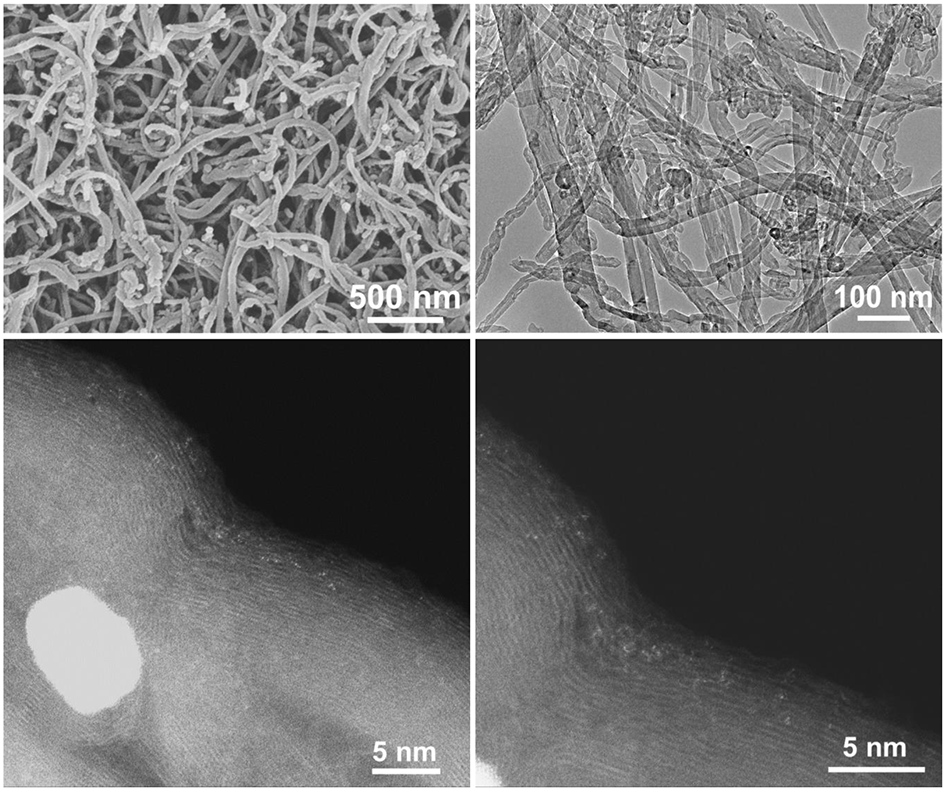


**a**

**b**

**c**

**d**

**Figure S17.** (a) SEM, (b) TEM and (c-d) aberration-corrected HAADF-STEM images of CoSAs/SNPs-OCNTs after consecutive cycling tests.


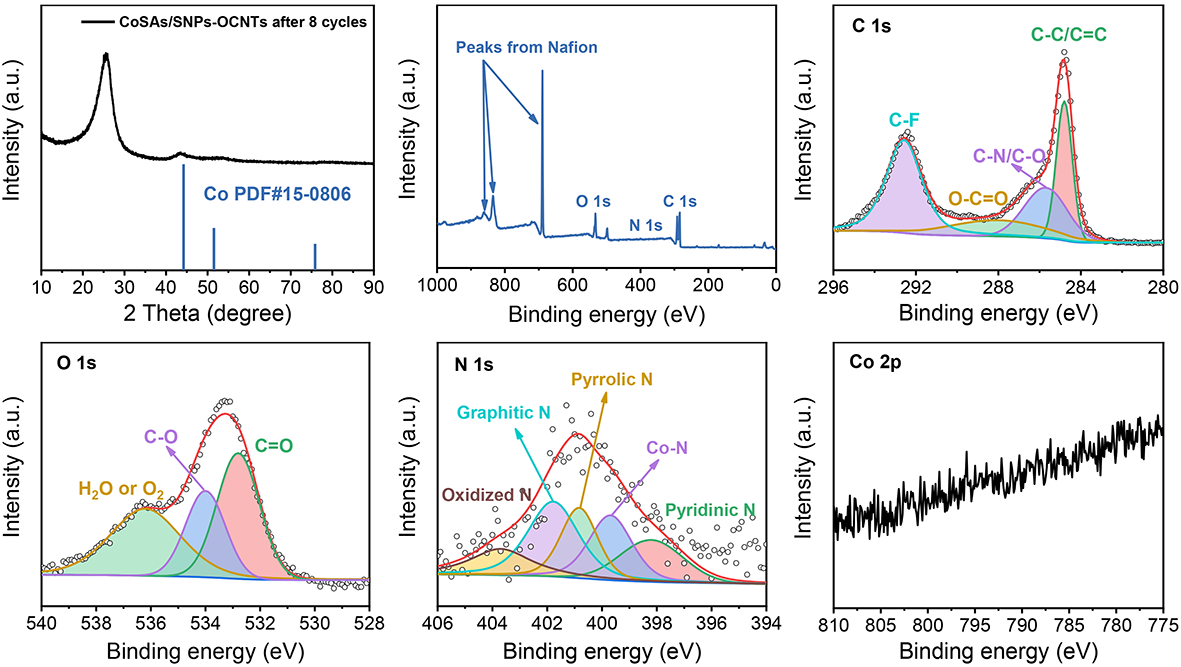


**a**

**d**

**b**

**e**

**c**

**f**

**Figure S18.** (a) XRD pattern and (b) XPS survey spectrum of CoSAs/SNPs-OCNTs after consecutive cycling tests. High-resolution (c) C1s, (d) O 1s (e) N 1s and (e) Co 2p XPS spectra of CoSAs/SNPs-OCNTs after consecutive cycling tests. Note that the additional peaks are from Nafion, which was added as the binder during the preparation of catalyst ink.

**a**

**b**

**
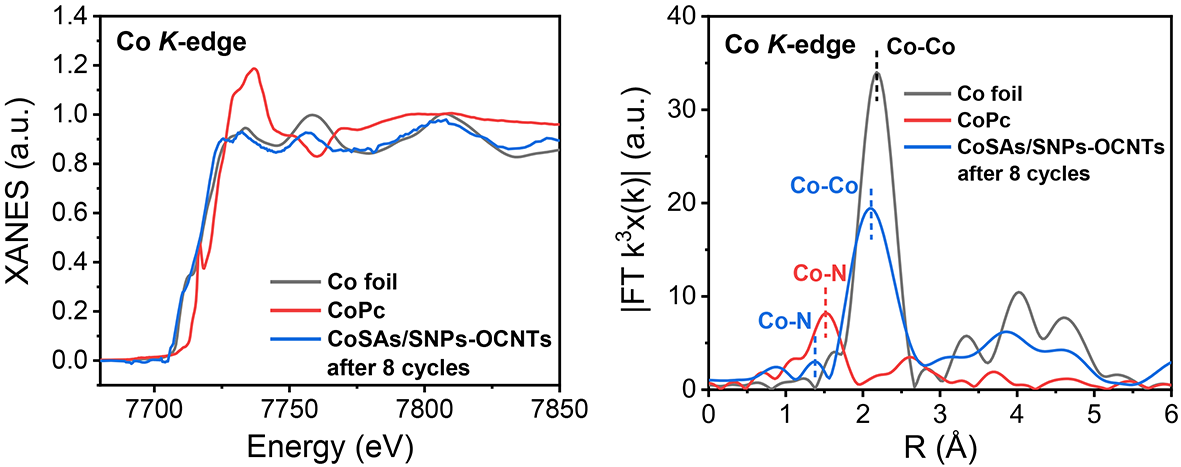
**

**Figure S19.** Co K-edge (a) XANES spectra and (b) k^3^-weighted FT-EXAFS spectra of CoSAs/SNPs-OCNTs after 8 cycles and references.


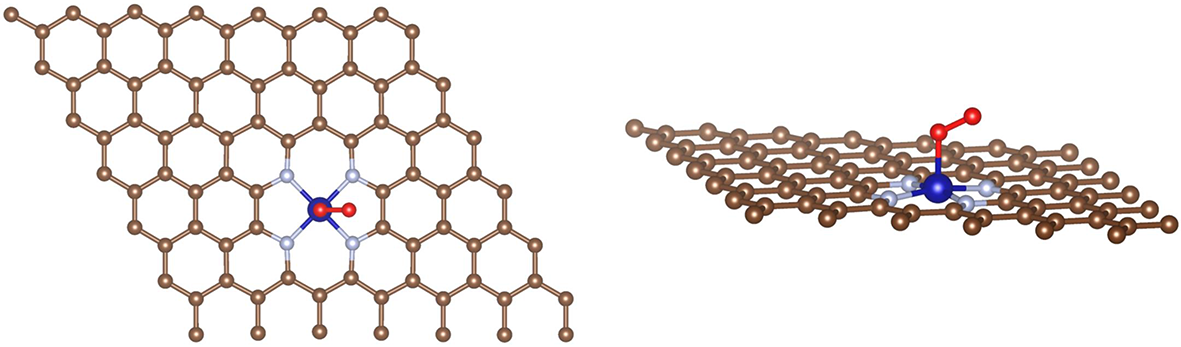


**a**

**b**

**Figure S20.** (a) Top view and (b) side view of the optimized structures of *O_2_ adsorption on Co-N_4_/C. Brown sphere: carbon, blue sphere: cobalt, wathet sphere: nitrogen, red sphere: oxygen.


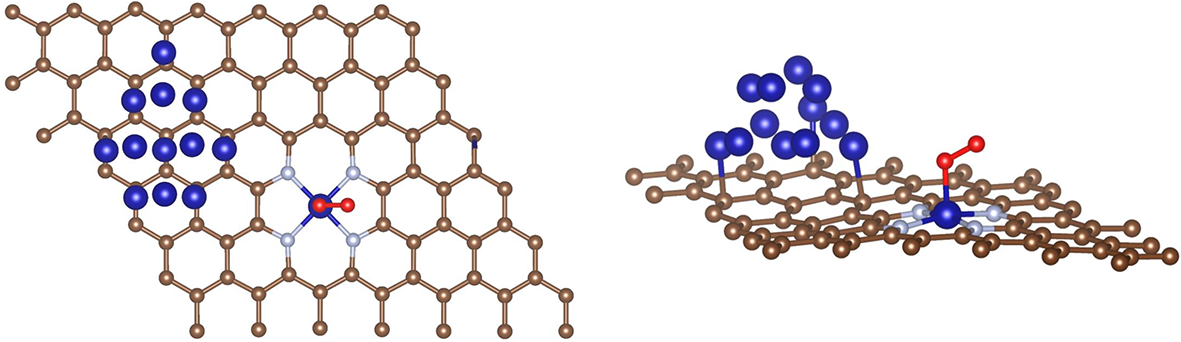


**a**

**b**

**Figure S21.** (a) Top view and (b) side view of the optimized structures of *O_2_ adsorption on Co-N_4_/Co_12_/C. Brown sphere: carbon, blue sphere: cobalt, wathet sphere: nitrogen, red sphere: oxygen.


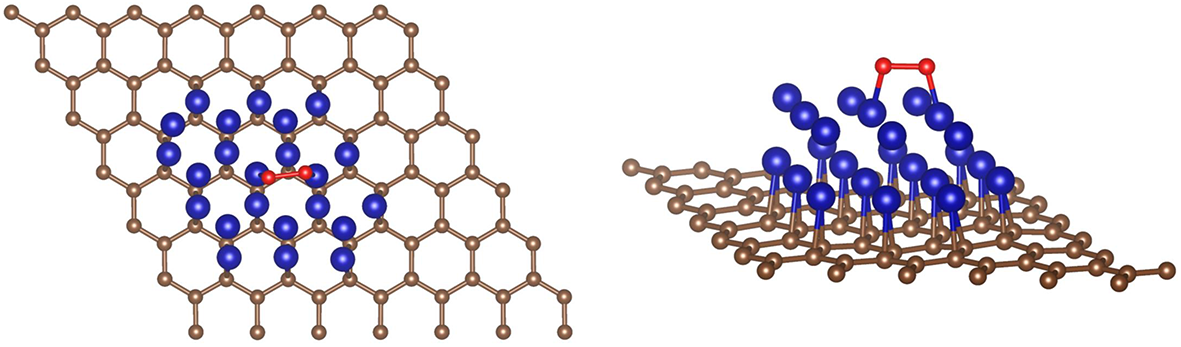


**a**

**b**

**Figure S22.** (a) Top view and (b) side view of the optimized structures of *O_2_ adsorption on Co_23_/C. Brown sphere: carbon, blue sphere: cobalt, wathet sphere: nitrogen, red sphere: oxygen.


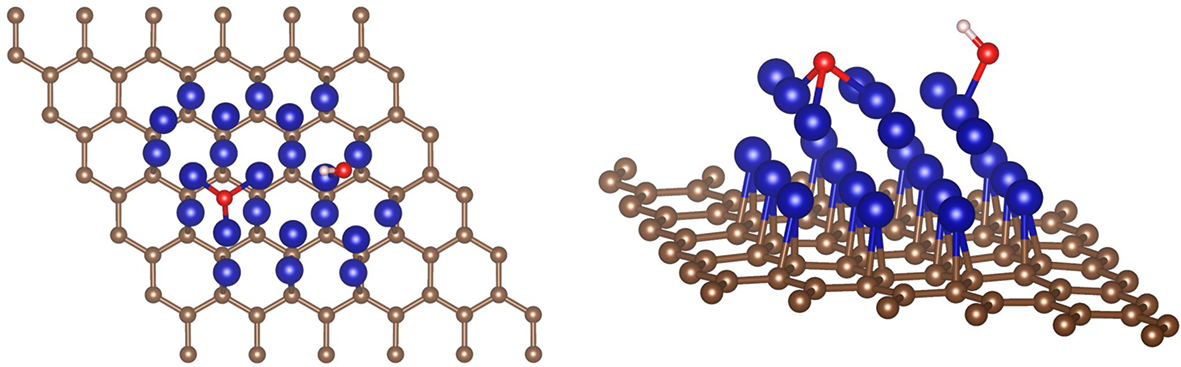


**a**

**b**

**Figure S23.** (a) Top view and (b) side view of the optimized structures of *OOH dissociating to *O and *OH on Co_23_/C. Brown sphere: carbon, blue sphere: cobalt, red sphere: oxygen, white sphere: hydrogen.


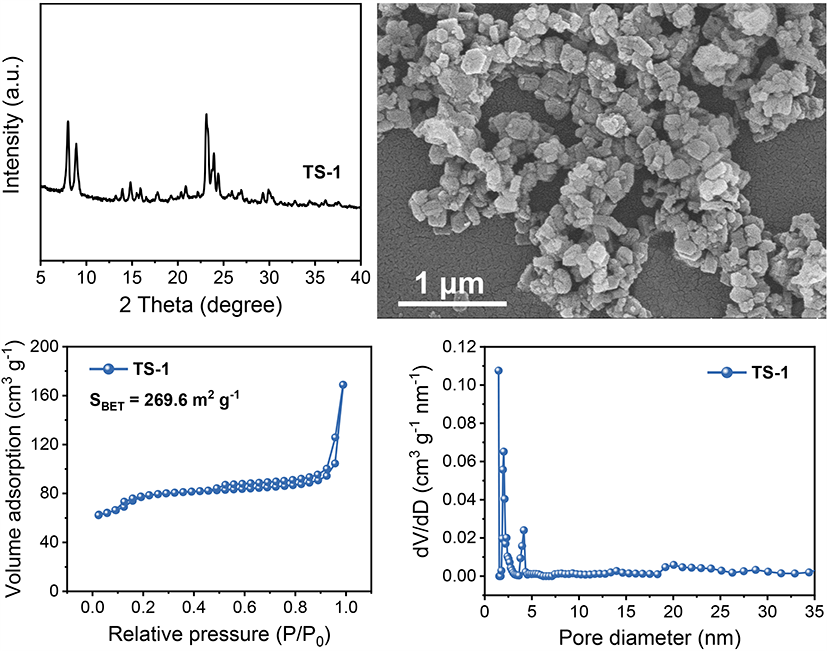


**a**

**b**

**c**

**d**

**Figure S24.** (a) XRD pattern and (b) SEM image of TS-1. (c) Nitrogen adsorption-desorption isotherms of TS-1. (d) Corresponding pore size distribution curve.

**
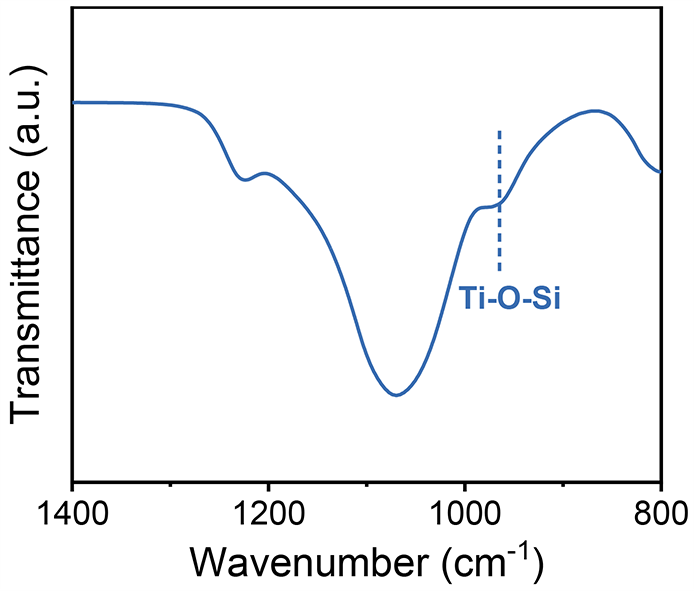
**

**Figure S25.** FT-IR spectrum of TS-1.

**
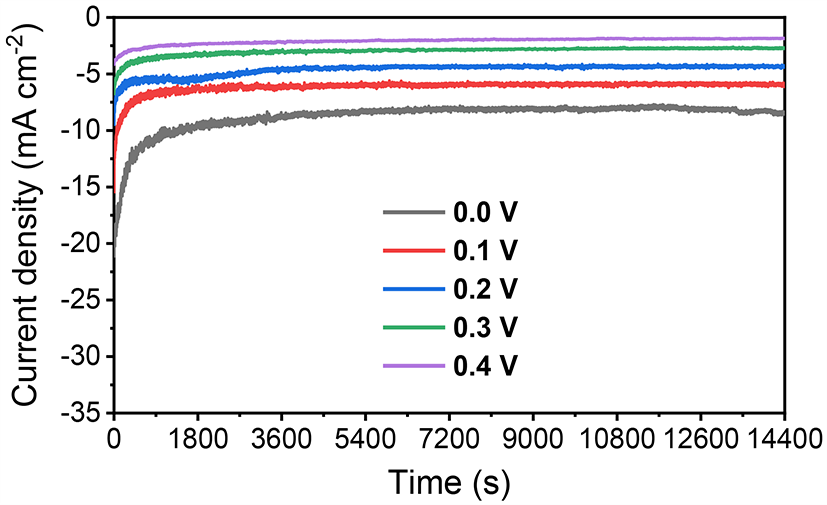
**

**Figure S26.** Chronoamperometric curves of CoSAs/SNPs-OCNTs operated at various applied potentials in O_2_-saturated 0.1 M NaPi containing TS-1, NH_3_·H_2_O and CYC.

**
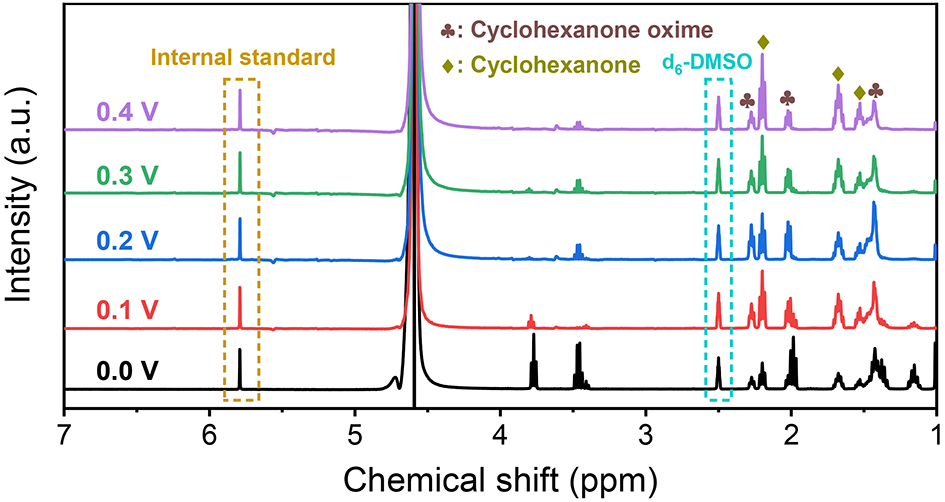
**

**Figure S27.** ^1^H NMR spectra of the catholyte after electrolysis at different applied potentials for 4 h.


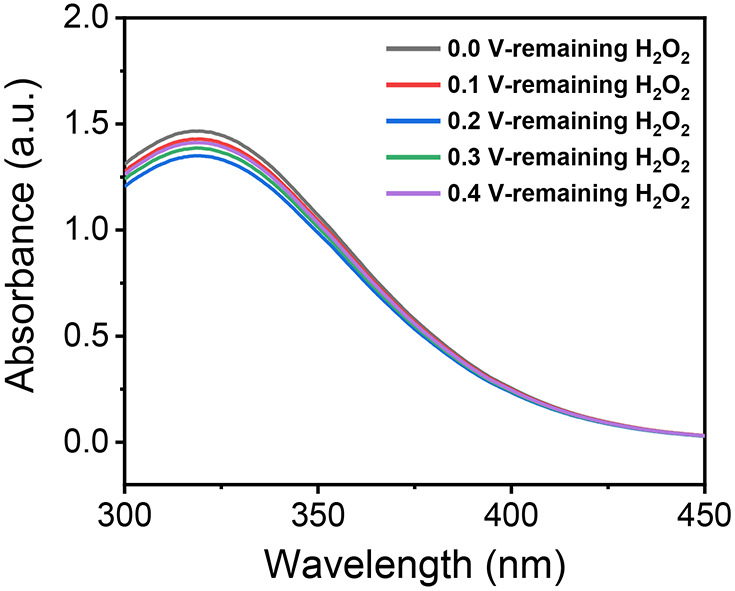


**Figure S28.** UV-vis absorption spectra of Ce(SO_4_)_2_ solutions after injecting 50 μL of electrolytes after ammoximation reaction into 3.0 mL of 0.5 mM Ce(SO_4_)_2_ solutions.


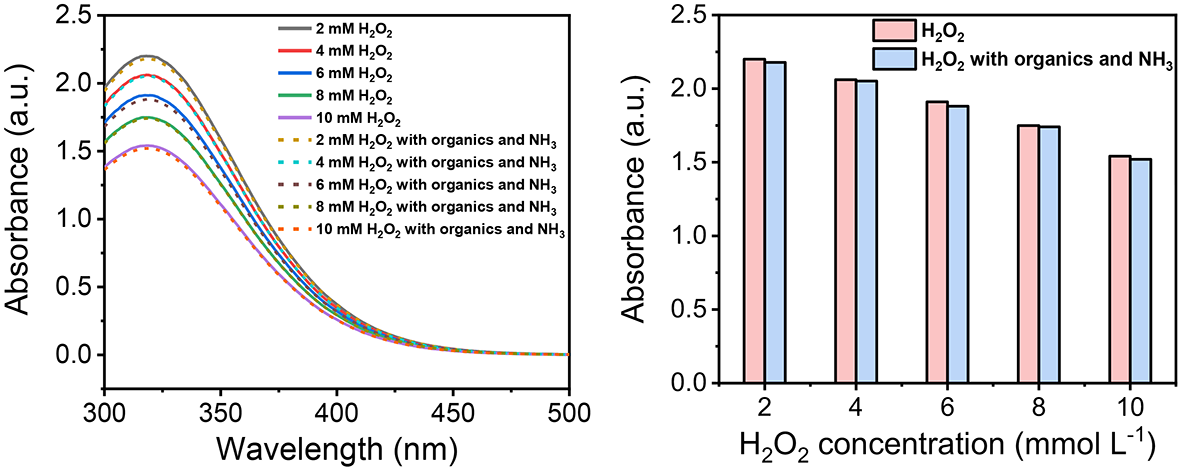


**a**

**b**

**Figure S29.** (a) UV-vis absorption spectra of Ce(SO_4_)_2_ solutions after injecting 50 μL of electrolytes containing different H_2_O_2_ concentrations with or without organics and ammonia into 3.0 mL of 0.5 mM Ce(SO_4_)_2_ solutions. (b) Comparison of absorbance values at 319 nm of Ce(SO_4_)_2_ solutions containing different H_2_O_2_ concentrations with or without organics and ammonia. To simulate the electrolyte composition after the ammoximation process, 5 mM cyclohexanone, 5 mM cyclohexanone oxime and 100 mM ammonia were added into a 0.1 M NaPi solution.

**
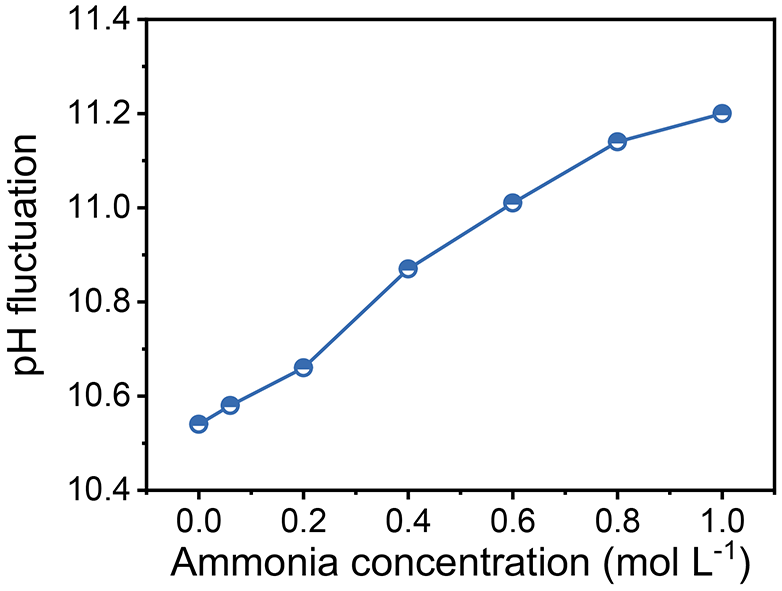
**

**Figure S30.** pH fluctuation with the ammonia concentration in the catholyte.

**
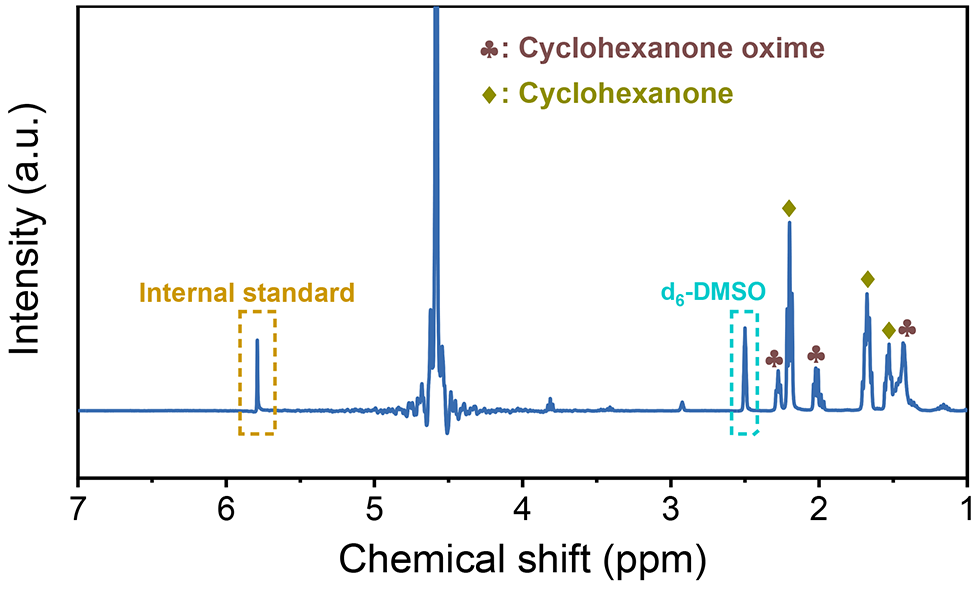
**

**Figure S31.** ^1^H NMR spectra of the solution after ammoximation reaction for 4 h in the commercial H_2_O_2_ system. The H_2_O_2_ concentration was maintained at 20 mM, which is similar to the concentration generated by the CoSAs/SNPs-OCNTs catalyst during the 2e^-^ ORR process.

**
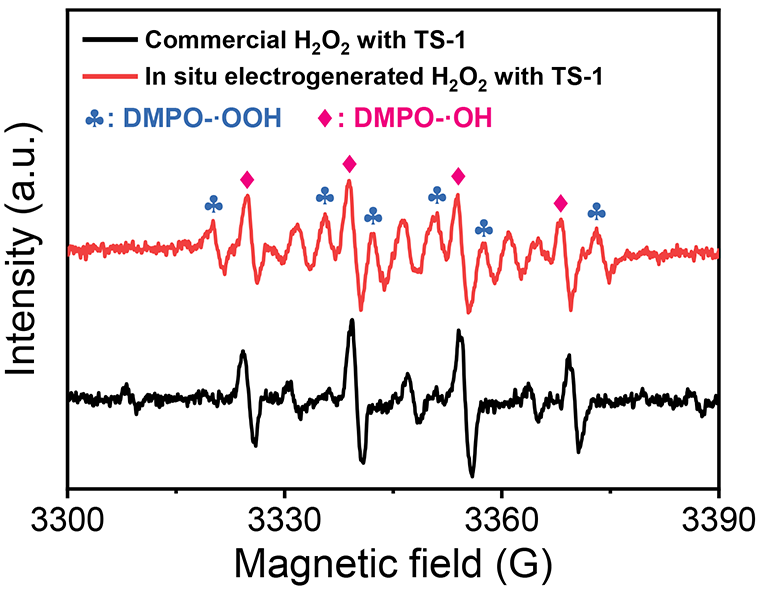
**

**Figure S32.** DMPO spin-trapping EPR spectra in commercial H_2_O_2_ system and *in situ* electrogenerated H_2_O_2_ system.


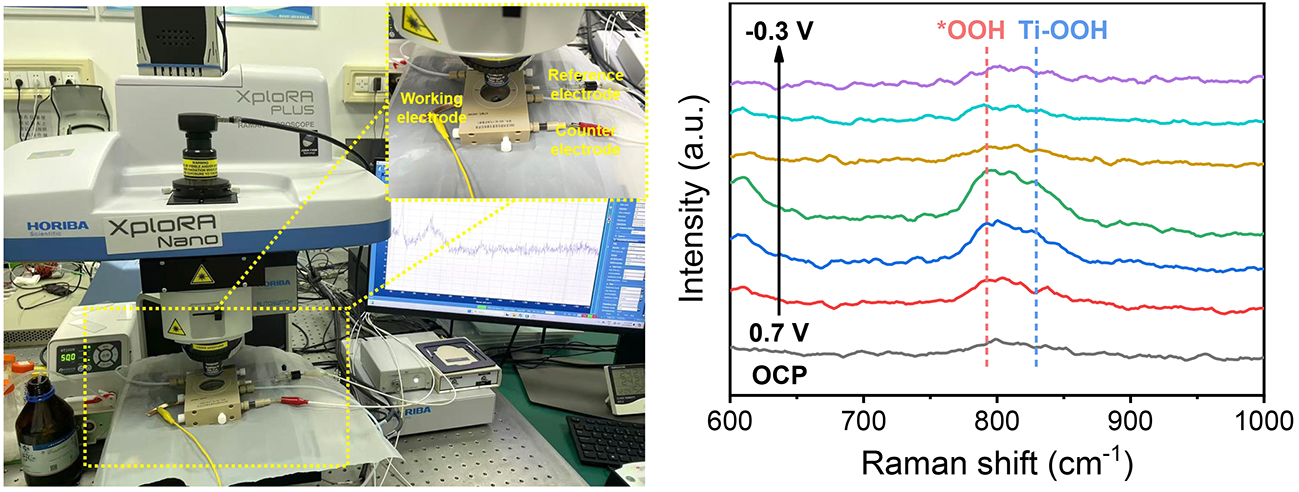


**a**

**b**

**Figure S33.** (a) The optical photograph of *in situ* Raman spectroscopy set-up. (b) *In situ* Raman spectra in *in situ* electrogenerated H_2_O_2_ coupling CYC ammoximation system.


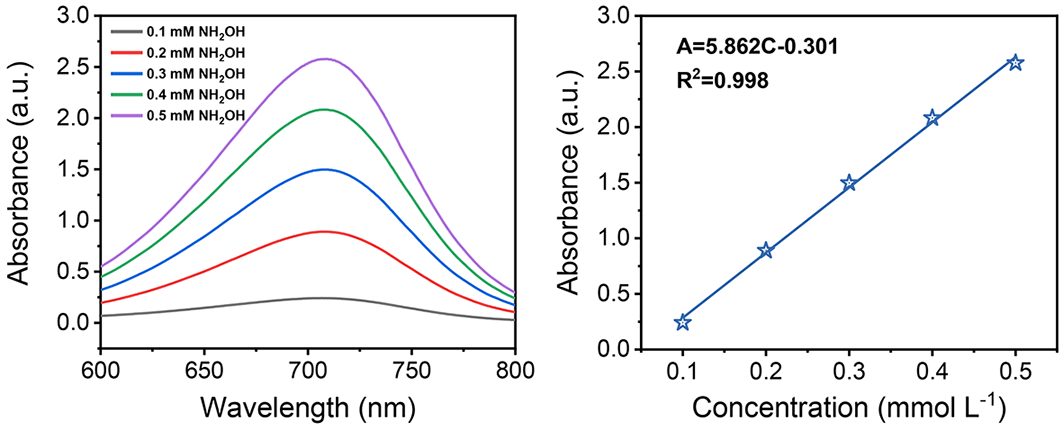


**a**

**b**

**Figure S34.** Quantification of NH_2_OH by the spectrophotometric method. (a) Corresponding UV-vis absorption spectra. (b) Linear peak absorbance-concentration calibration curve at 708 nm.

**
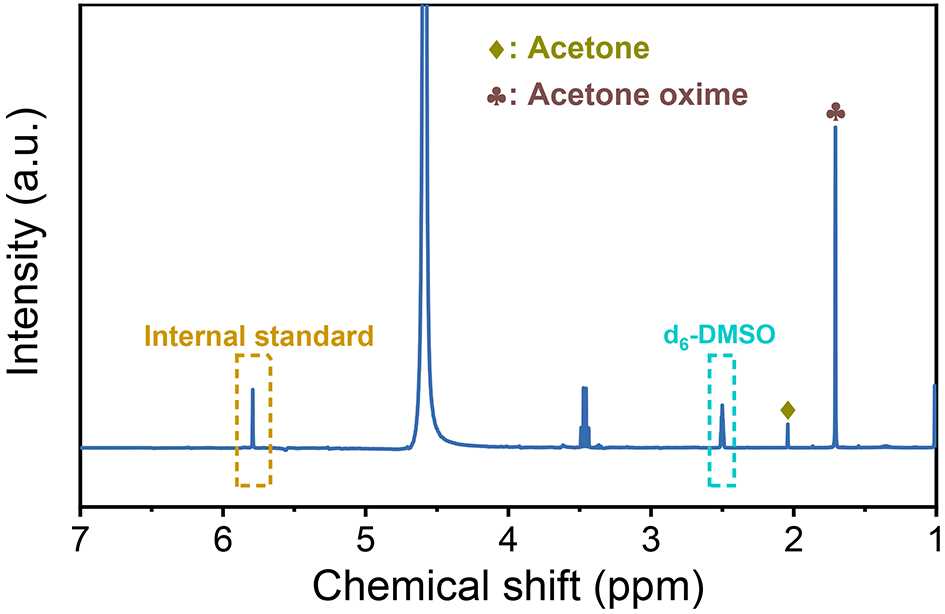
**

**Figure S35.** ^1^H NMR spectra of the catholyte after electrolysis at 0.2 V (*vs.* RHE) for 4 h with acetone as the substrate.

**
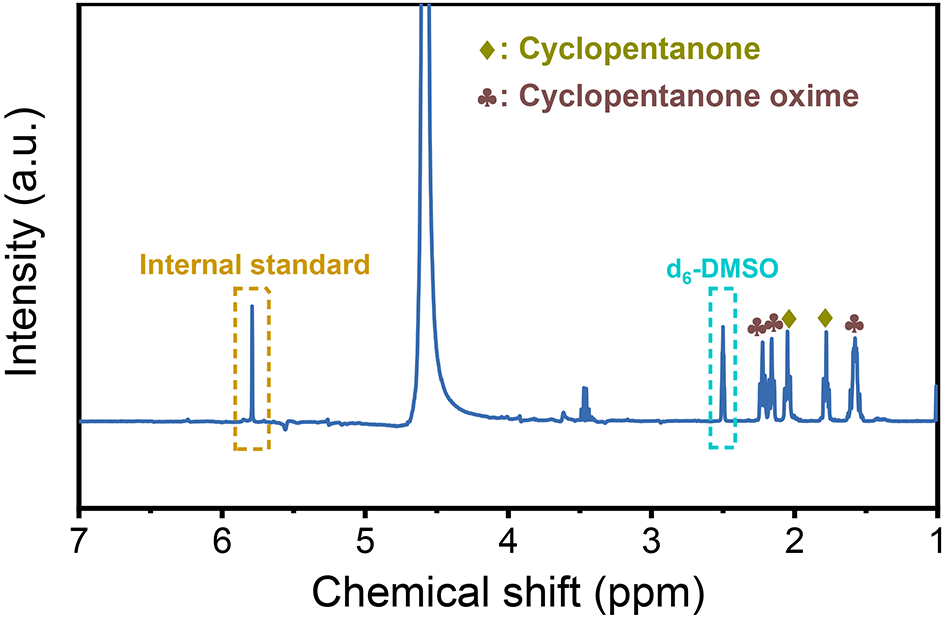
**

**Figure S36.** ^1^H NMR spectra of the catholyte after electrolysis at 0.2 V (*vs.* RHE) for 4 h with cyclopentanone as the substrate.

**
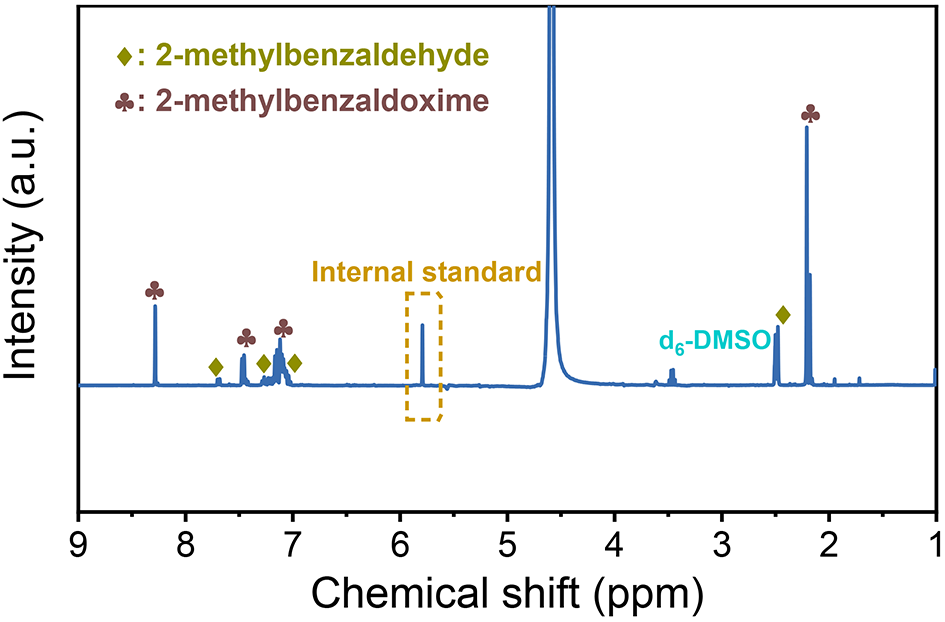
**

**Figure S37.** ^1^H NMR spectra of the catholyte after electrolysis at 0.2 V (*vs.* RHE) for 4 h with 2-methylbenzaldehyde as the substrate.

**
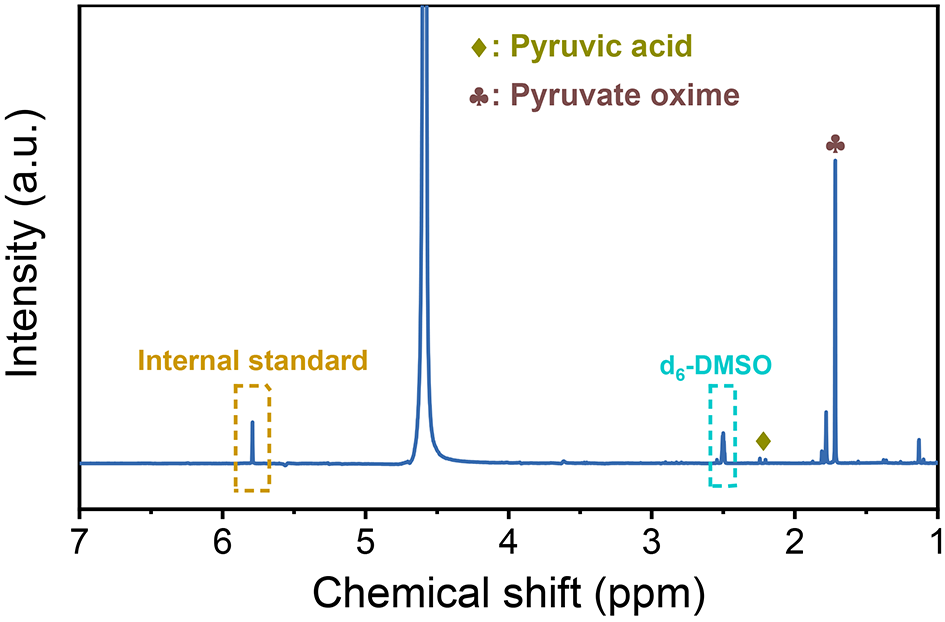
**

**Figure S38.** ^1^H NMR spectra of the catholyte after electrolysis at 0.2 V (*vs.* RHE) for 4 h with pyruvic acid as the substrate.

**
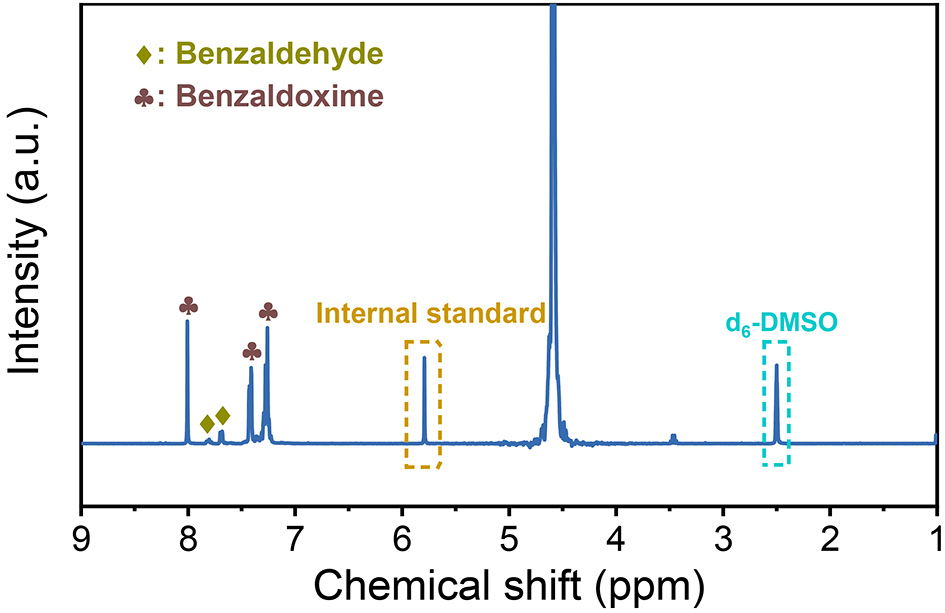
**

**Figure S39.** ^1^H NMR spectra of the catholyte after electrolysis at 0.2 V (*vs.* RHE) for 4 h with benzaldehyde as the substrate.

**Table S1.** Comparison of our work and recently reported single atom electrocatalysts for 2e^-^ ORR.

| **Catalyst** | **Electrolyte** | **Onset potential (V *vs.* RHE)** | **Selectivity (%)** | **Ref.** |
| --- | --- | --- | --- | --- |
| Co-POC-O | 0.1 M KOH | 0.84 | 85.6 | 8 |
| Co_1_@GO | 0.1 M KOH | 0.91 | 81.4 | 9 |
| Co_1_-NG(O) | 0.1 M KOH | ~0.83 | 82 | 10 |
| Co-N_2_-C/HO | 0.1 M KOH | 0.801 | 91.3 | 11 |
| Co/NC | 0.1 M PBS | ~0.80 | 90 | 12 |
| CoN_4_C-N | 0.1 M HClO_4_ | ~0.70 | 92 | 13 |
| FeN_2_O_2_ | 0.1 M KOH | 0.79 | 95 | 14 |
| Fe_SA_-NS/C-700 | 0.1 M KOH | 0.84 | 92 | 15 |
| Mo_1_/OSG-H | 0.1 M KOH | 0.78 | 95 | 16 |
| Ni-N_2_O_2_/C | 0.1 M KOH | ~0.70 | 96 | 17 |
| NiN_x_/C-AQNH_2_ | 0.1 M KOH | 0.81 | 80 | 18 |
| NiOC | 0.1 M PBS | ~0.50 | 90 | 19 |
| In SAs/NSBC | 0.1 M KOH | ~0.80 | 95.4 | 20 |
| W_1_/NO-C | 0.1 M KOH | 0.815 | 90 | 21 |
| ZnO_3_C | 0.1 M KOH | ~0.80 | 89 | 22 |
| O-C(Al) | 0.1 M KOH | 0.822 | 95 | 23 |
| **CoSAs/SNPs-OCNTs** | **0.1 M NaPi** | **0.73** | **95.9** | **This work** |

**Table S2.** List of control experiments.

| **Entry** | **C source** | **N source** | **O source** | **Heterogeneous catalyst** | **Electricity** | **CHO?** |
| --- | --- | --- | --- | --- | --- | --- |
| 1 | CYC | NH_3_·H_2_O | O_2_ | TS-1 | Yes | Yes |
| 2 | CYC | NH_3_·H_2_O | O_2_ | - | Yes | No |
| 3 | CYC | NH_3_·H_2_O | - | TS-1 | Yes | No |
| 4 | CYC | NH_3_·H_2_O | O_2_ | TS-1 | No | No |
| 5 | CYC | - | O_2_ | TS-1 | Yes | No |
| 6 | CYC | NH_2_OH | - | - | No | Yes |

**Table S3.** Comparison of our work and other recently reported catalysts for electrochemical synthesis of cyclohexanone oxime.

| **Catalyst** | **Electrolyte** | **N source** | **Yield rate** | **FE (%)** | **Ref.** |
| --- | --- | --- | --- | --- | --- |
| CP/PTFE | 0.25 M Li_2_SO_4_ | NO | 10.7 mg cm^-2^ h^-1^ | 44.8 | 24 |
| Cu-S | 0.5 M PBS | NaNO_2_ | 0.165 mmol cm^-2^ h^-1^ | 26 | 25 |
| Fe | 0.5 M K_2_CO_3_ | KNO_3_ | 59.5 g h^-1^ g_cat_^-1^  (flow cell) | ~20 | 26 |
| Zn_93_Cu_7_ | 0.5 M KPi | KNO_3_ | - | 27 | 27 |
| R-TiO_2_ | 0.5 M Na_2_CO_3_-NaHCO_3_ | KNO_3_ | 0.127 mmol cm^-2^ h^-1^ | 68.2 | 28 |
| **CoSAs/SNPs-OCNTs** | **0.1 M NaPi** | **NH_3_·H_2_O** | **75.6±0.5 mmol g_ecat_^-1^ h^-1^** | **72.0±1.9** | **This work** |

**References**

[1] a) G. Kresse, J. Hafner, *Phys. Rev. B* **1994**, *49*, 14251-14269; b) G. Kresse, J. Furthmüller, *Phys. Rev. B* **1996**, *54*, 11169-11186; c) G. Kresse, J. Furthmüller, *Comput. Mater. Sci.* **1996**, *6*, 15-50.

[2] P. E. Blöchl, *Phys. Rev. B* **1994**, *50*, 17953-17979.

[3] J. P. Perdew, K. Burkeand, M. Ernzerhof, *Phys. Rev. Lett.* **1996**, *77*, 3865-3868.

[4] S. Grimme, J. Antony, S. Ehrlich, H. Krieg, *J. Chem. Phys.* **2010**, *132*, 154104.

[5] V. L. Deringer, A. L. Tchougreeff, R. Dronskowski, *J. Phys. Chem. A* **2011**, *115*, 5461-5466.

[6] a) S. Siahrostami, A. Verdaguer-Casadevall, M. Karamad, D. Deiana, P. Malacrida, B. Wickman, M. Escudero-Escribano, E. A. Paoli, R. Frydendal, T. W. Hansen, I. Chorkendorff, I. E. Stephens, J. Rossmeisl, *Nat. Mater.* **2013**, *12*, 1137-1143; b) A. Verdaguer-Casadevall, D. Deiana, M. Karamad, S. Siahrostami, P. Malacrida, T. W. Hansen, J. Rossmeisl, I. Chorkendorff, I. E. Stephens, *Nano Lett.* **2014**, *14*, 1603-1608; c) J. S. Jirkovsky, I. Panas, E. Ahlberg, M. Halasa, S. Romani, D. J. Schiffrin, *J. Am. Chem. Soc.* **2011**, *133*, 19432-19441.

[7] a) J. Park, Y. Nabae, T. Hayakawa, M.-a. Kakimoto, *ACS Catal.* **2014**, *4*, 3749-3754; b) H. W. Kim, M. B. Ross, N. Kornienko, L. Zhang, J. Guo, P. Yang, B. D. McCloskey, *Nat. Catal.* **2018**, *1*, 282-290.

[8] B. Q. Li, C. X. Zhao, J. N. Liu, Q. Zhang, *Adv. Mater.* **2019**, *31*, 1808173.

[9] B. W. Zhang, T. Zheng, Y. X. Wang, Y. Du, S. Q. Chu, Z. Xia, R. Amal, S. X. Dou, L. Dai, *Commun. Chem.* **2022**, *5*, 43.

[10] E. Jung, H. Shin, B. H. Lee, V. Efremov, S. Lee, H. S. Lee, J. Kim, W. Hooch Antink, S. Park, K. S. Lee, S. P. Cho, J. S. Yoo, Y. E. Sung, T. Hyeon, *Nat. Mater.* **2020**, *19*, 436-442.

[11] H. Gong, Z. Wei, Z. Gong, J. Liu, G. Ye, M. Yan, J. Dong, C. Allen, J. Liu, K. Huang, R. Liu, G. He, S. Zhao, H. Fei, *Adv. Funct. Mater.* **2021**, *32*, 2106886.

[12] H. Shen, N. Qiu, L. Yang, X. Guo, K. Zhang, T. Thomas, S. Du, Q. Zheng, J. P. Attfield, Y. Zhu, M. Yang, *Small* **2022**, 18, e2200730.

[13] W. Wang, Y. Hu, P. Li, Y. Liu, S. Chen, *ACS Catal.* **2024**, 14, 5961-5971.

[14] Y. Wu, Y. Ding, X. Han, B. Li, Y. Wang, S. Dong, Q. Li, S. Dou, J. Sun, J. Sun, *Appl. Catal. B Environ.* **2022**, *315*, 121578.

[15] Y. Li, J. Chen, Y. Ji, Z. Zhao, W. Cui, X. Sang, Y. Cheng, B. Yang, Z. Li, Q. Zhang, L. Lei, Z. Wen, L. Dai, Y. Hou, *Angew. Chem. Int. Ed.* **2023**, 62, e202306491.

[16] C. Tang, Y. Jiao, B. Shi, J. N. Liu, Z. Xie, X. Chen, Q. Zhang, S. Z. Qiao, *Angew. Chem. Int. Ed.* **2020**, *59*, 9171-9176.

[17] Y. Wang, R. Shi, L. Shang, G. I. N. Waterhouse, J. Zhao, Q. Zhang, L. Gu, T. Zhang, *Angew. Chem. Int. Ed.* **2020**, *59*, 13057-13062.

[18] X. Li, S. Tang, S. Dou, H. J. Fan, T. S. Choksi, X. Wang, *Adv. Mater.* **2022**, *34*, 2104891.

[19] W. Xu, Z. Liang, S. Gong, B. Zhang, H. Wang, L. Su, X. Chen, N. Han, Z. Tian, T. Kallio, L. Chen, Z. Lu, X. Sun, *ACS Sustainable Chem. Eng*. **2021**, 9, 7120-7129.

[20] E. Zhang, L. Tao, J. An, J. Zhang, L. Meng, X. Zheng, Y. Wang, N. Li, S. Du, J. Zhang, D. Wang, Y. Li, *Angew. Chem. Int. Ed.* **2022**, *61*, e202117347.

[21] F. Zhang, Y. Zhu, C. Tang, Y. Chen, B. Qian, Z. Hu, Y. C. Chang, C. W. Pao, Q. Lin, S. A. Kazemi, Y. Wang, L. Zhang, X. Zhang, H. Wang, *Adv. Funct. Mater.* **2021**, *32*, 2110224.

[22] Y. Jia, Z. Xue, J. Yang, Q. Liu, J. Xian, Y. Zhong, Y. Sun, X. Zhang, Q. Liu, D. Yao, G. Li, *Angew. Chem. Int. Ed.* **2022**, *61*, e202110838.

[23] Q. Yang, W. Xu, S. Gong, G. Zheng, Z. Tian, Y. Wen, L. Peng, L. Zhang, Z. Lu, L. Chen, *Nat. Commun.* **2020**, *11*, 5478.

[24] X. Zhang, H. Jing, S. Chen, B. Liu, L. Yu, J. Xiao, D. Deng, *Chem Catal.* **2022**, 2, 1807-1818.

[25] Y. Wu, J. Zhao, C. Wang, T. Li, B. H. Zhao, Z. Song, C. Liu, B. Zhang, *Nat. Commun.* **2023**, 14, 3057.

[26] Y. Wu, W. Chen, Y. Jiang, Y. Xu, B. Zhou, L. Xu, C. Xie, M. Yang, M. Qiu, D. Wang, Q. Liu, Q. Liu, S. Wang, Y. Zou, *Angew. Chem. Int. Ed.* **2023**, 62, e202305491.

[27] J. Sharp, A. Ciotti, H. Andrews, S. R. Udayasurian, M. García-Melchor, T. Li, *ACS Catal.* **2024**, 14, 3287-3297.

[28] L. Luo, L. Li, L. Xu, Y. Yan, S. Zhang, H. Zhou, Z. Li, M. Shao, X. Duan, *CCS Chem.* **2024**, 1-13.
